# A single amino acid in the *Salmonella* effector SarA/SteE triggers supraphysiological activation of STAT3 for anti-inflammatory gene expression

**DOI:** 10.1016/j.celrep.2025.115530

**Published:** 2025-04-05

**Authors:** Margaret R. Gaggioli, Angela G. Jones, Ioanna Panagi, Erica J. Washington, Rachel E. Loney, Janina H. Muench, Matthew W. Foster, Richard G. Brennan, Teresa L.M. Thurston, Dennis C. Ko

**Affiliations:** 1 Department of Molecular Genetics and Microbiology, School of Medicine, Duke University, Durham, NC 27710, USA; 2 Department of Infectious Disease, Centre for Bacterial Resistance Biology, Imperial College London, London, UK; 3 Department of Biochemistry, School of Medicine, Duke University, Durham, NC 27710, USA; 4 The Francis Crick Institute, NW1 1AT London, UK; 5 Duke Proteomics and Metabolomics Core Facility, School of Medicine, Duke University, Durham, NC 27710, USA; 6 Division of Infectious Diseases, Department of Medicine, School of Medicine, Duke University, Durham, NC 27710, USA

**Keywords:** GSK-3, STM2585, gogC, JAK-STAT, SOCS3, IL6ST, Y705, IL-6, IL-10, adaptation

## Abstract

*Salmonella* causes ∼1 million cases of gastroenteritis annually in the United States. Critical to virulence are secreted effectors that reprogram host functions. We previously discovered the effector SarA facilitates phosphorylation of STAT3, inducing expression of the anti-inflammatory cytokine interleukin-10 (IL-10). This STAT3 activation requires a region of homology with the host cytokine receptor gp130. Here, we demonstrate that a single amino acid difference is critical for the anti-inflammatory bias of SarA-STAT3 signaling. An isoleucine at pY+1 of the YxxQ motif in SarA (which binds the STAT3 SH2 domain) causes increased STAT3 recruitment and phosphorylation, biasing toward anti-inflammatory targets. This isoleucine renders SarA a better substrate for tyrosine phosphorylation by GSK-3. GSK-3 is canonically a serine/threonine kinase that nonetheless undergoes tyrosine autophosphorylation at a motif with isoleucine at the pY+1 position. Our results provide a molecular basis for how a *Salmonella* effector achieves supraphysiological levels of STAT3 activation to control host genes.

## Highlights

• *Salmonella* effector SarA activates STAT3 biased toward anti-inflammatory targets
• Anti-inflammatory bias is due to stronger SarA binding and phosphorylation of STAT3
• Isoleucine-168 makes SarA a better substrate for tyrosine phosphorylation by GSK-3
• Isoleucine-168 matches the GSK3 YIxQ autophosphorylation motif

## Graphical abstract

**Figure:**
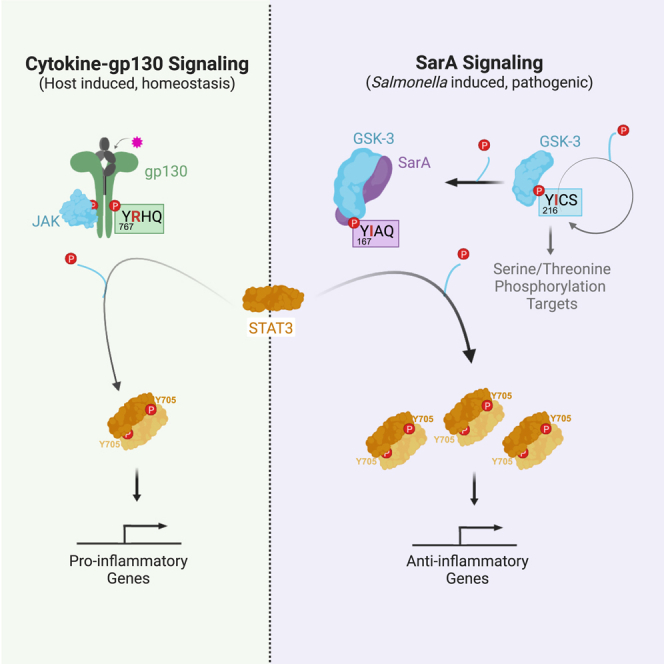

## Teaser

Gaggioli et al. perform a comparative study of STAT3 activation by the *Salmonella* effector SarA and the host cytokine co-receptor gp130. SarA activation is biased toward anti-inflammatory target genes because its STAT3 binding motif has evolved to match the tyrosine autophosphorylation motif of GSK-3.

## Introduction

*Salmonella enterica* serovar Typhimurium is a gram-negative facultative intracellular anaerobe and a causative agent of salmonellosis, a gastrointestinal infection that is one of the most common foodborne diseases in humans. Salmonellosis outbreaks are a major public health concern, with an estimated 1 million annual cases in the United States alone.^1^

Essential to the pathogenesis of *Salmonella* Typhimurium, and all *S. enterica* serovars, is an arsenal of secreted protein effectors.^2^ These effectors are translocated into the host cell by type III secretion systems (T3SS) encoded within *Salmonella* pathogenicity islands (SPI).^3^ Secreted protein effectors can mimic and reprogram host cellular functions to create a beneficial environment for the invading bacteria, such as formation of the *Salmonella-*containing vacuole and antagonization of the immune response.^4^ While secreted factors are important to all *Salmonella* serovars, different serovars have different repertoires of effectors, reflecting the diverse niches and pathogenic outcomes seen with *S. enterica*.

We previously used this natural diversity to identify the effector SarA (*Salmonella* anti-inflammatory response activator; also known as Stm2585, GogC, and SteE). SarA acts through the activation of the transcription factor signal transducer and activator of transcription 3 (STAT3) to regulate the host response to *Salmonella* Typhimurium infection.^5^ SarA is secreted primarily by the SPI-2 T3SS^5^ and recruits the kinase glycogen synthase kinase-3 (GSK-3), causing phosphorylation of SarA at a YxxQ motif.^6^^,^^7^ This phosphorylated motif binds STAT3, which in turn is also phosphorylated by GSK-3 at the critical tyrosine for STAT3 activation, Y705.^6^^,^^7^ Activation of STAT3 stimulates intracellular bacterial replication and production of the anti-inflammatory cytokine interleukin-10 (IL-10),^5^ as well as M2 polarization of macrophages.^7^^,^^8^^,^^9^

**Figure 1:**
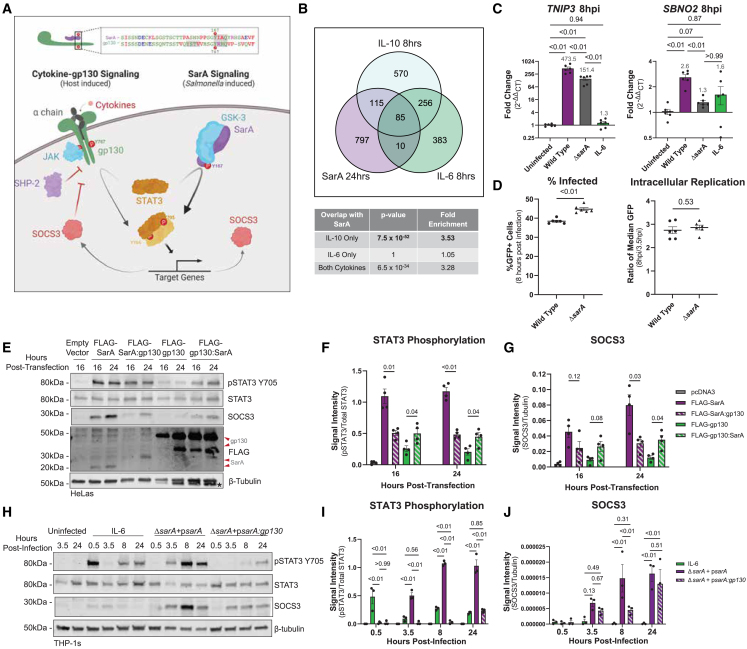
SarA induces anti-inflammatory cell signaling and strong STAT3 phosphorylation (A) Graphical comparison of SarA vs. gp130 activation of STAT3, including sequence alignment of SarA and gp130 GBS domain. Schematic created using BioRender. (B) SarA upregulated genes significantly overlap with IL-10 target genes. Venn diagrams showing overlap of genes upregulated in a SarA-dependent manner (at 24 h post-*Salmonella* Typhimurium infection in LCLs [data from Jaslow et al.^5^]) and in an IL-10- or IL-6-dependent manner (after 8 h cytokine stimulation in dendritic cells [data from Braun et al.^10^]). *p* values obtained from a chi-squared test. See also Figure S1. (C) Wild-type *Salmonella* Typhimurium infection leads to increased expression of anti-inflammatory genes. THP-1 cells were either stimulated with 10 ng/mL of IL-6 or infected with wild type or *ΔsarA Salmonella* Typhimurium. RNA was collected from cells 8 hpi, and expression levels of *SBNO2* and *TNIP3* were measured via qPCR. Points represent six biological replicates across three experiments (with each biological replicate shown being the average of three qPCR technical replicates). Gray values above bars are the mean. Data are represented as mean ± SEM. *p* values obtained from Brown-Forsythe and Welch ANOVAs with Dunnett’s T3 multiple comparisons test. (D) *ΔsarA* mutant strain of *Salmonella* Typhimurium has a significantly higher percentage of infection at 8 hpi, but wild type and *ΔsarA* have similar rates of intracellular replication. THP-1 cells were infected at MOI 10 with inducible GFP-expression bacteria. Bacterial replication was quantified as the ratio of median GFP value of infected cells at 8 hpi over 3.5 hpi. Points represent six biological replicates across three independent experiments. Data are represented as mean ± SEM. *p* values obtained from unpaired t tests with Welch’s correction. (E) SarA GBS domain leads to greater STAT3 phosphorylation and SOCS3 abundance compared to gp130 GBS domain as quantified by western blot after overexpression in HeLa cells. The asterisk on the β-tubulin blot marks the residual signal from the ∼50-kDa FLAG band of FLAG-gp130; they are similar molecular weights, and both were detected using anti-mouse secondary antibody. Representative of four experiments that are quantified in (F) and (G). See also Figure S3. (F) Quantification of STAT3 phosphorylation at each time point from western blot in (E). Points represent four experiments. Data are represented as mean ± SEM. *p* values obtained from unpaired t tests with Welch’s correction comparing FLAG-SarA to FLAG-SarA:gp130 and FLAG-gp130 to FLAG-gp130:SarA. (G) Quantification of SOCS3 abundance at each time point from western blot in (E). Points represent four experiments. Data are represented as mean ± SEM. *p* values obtained from unpaired t tests, with Welch’s correction comparing FLAG-SarA to FLAG-SarA:gp130 and FLAG-gp130 to FLAG-gp130:SarA. (H) SarA GBS domain leads to greater STAT3 phosphorylation and SOCS3 abundance compared to gp130 GBS domain as quantified by western blot after *Salmonella* Typhimurium infection in THP-1 cells. Representative of four experiments that are quantified in (I) and (J). See also Figures S2 and S4. (I) Quantification of STAT3 phosphorylation at each time point from western blot in (H). Points represent four experiments. Data are represented as mean ± SEM. *p* values obtained from a two-way ANOVA with Tukey’s multiple comparisons test. (J) Quantification of SOCS3 abundance at each time point from western blot in (H). Points represent four experiments. Data are represented as mean ± SEM. *p* values obtained from a two-way ANOVA with Tukey’s multiple comparisons test.

The YxxQ motif and surrounding amino acids in SarA have similarity with the host cytokine co-receptor gp130 (Figure 1A).^6^ Paradoxically, gp130 is the shared signal transducing subunit for the pro-inflammatory IL-6 family of cytokines, yet SarA activation of STAT3 leads to production of the anti-inflammatory IL-10 (which is itself a stimulator of STAT3 activation). Studies comparing pro- and anti-inflammatory STAT3 signaling pathways support the idea that regulation of the strength and duration of STAT3 activation impacts downstream transcriptional targets.^10^^,^^11^^,^^12^^,^^13^^,^^14^ IL-6 stimulation of human cell cultures leads to a sharp, transient increase in STAT3 phosphorylation, while more robust and prolonged phosphorylation of STAT3 has been observed with IL-10 stimulation. Furthermore, prolonging phosphorylation through deletion of negative regulators^11^^,^^12^ or use of constitutively active gp130 dimers and STAT3^13^^,^^15^ transforms the transcriptional response induced upon IL-6 stimulation to become more similar to that induced by IL-10. These results suggest that the nature of STAT3-induced transcriptional changes is dictated by the strength and duration of phosphorylation. Therefore, we hypothesized that there are molecular characteristics of SarA-directed phosphorylation of STAT3 that make it more like anti-inflammatory activation than pro-inflammatory activation.

Here, we compared STAT3 activation mediated by SarA vs. gp130 to understand the nature and molecular basis of SarA-induced activation on the host transcriptional response. In response to SarA transfection or during infection, we observed greater phosphorylation of STAT3 and upregulation of anti-inflammatory genes. We hypothesized that more robust and prolonged activation by SarA could be due to either increased STAT3 binding or a lack of the negative regulation that canonically dampens gp130 signaling. Our results demonstrate that the amino acid residue at the pY+1 position of the SarA STAT3 binding YxxQ motif is critical for the increased binding of STAT3 to SarA compared to gp130. Unexpectedly, this effect is mediated through increased tyrosine phosphorylation of the SarA YxxQ motif by GSK-3, rather than an increased binding affinity to STAT3. The isoleucine at this position in SarA is invariant across 4,554 *S. enterica* sequences and matches the isoleucine conserved across 245 homologs in the GSK-3 autophosphorylated site (which has the sequence YICS). Thus, while SarA shares a 39-amino acid region of sequence similarity with gp130, SarA promotes supraphysiological activation of STAT3 because of a single amino acid difference that matches the substrate specificity of GSK-3, a host kinase with high baseline activity.

## Results

### SarA activation of STAT3 demonstrates an anti-inflammatory bias

The STAT3 signaling pathway can be activated by a wide variety of signals leading to either pro- or anti-inflammatory transcriptional responses. SarA was named as such because of its induction of IL-10 and other anti-inflammatory genes, but we also observed induction of pro-inflammatory genes such as *IFNG* and *CXCL9* in our previously published transcriptomics dataset comparing lymphoblastoid cell lines (LCLs; immortalized B cells) infected with wild type or *ΔsarA Salmonella* Typhimurium.^5^ Therefore, to better characterize the nature of SarA-dependent activation of STAT3, we compared our transcriptomics data to publicly available data generated from human monocyte-derived dendritic cells stimulated with either IL-6 or IL-10.^10^ We determined that genes activated by SarA during *Salmonella* Typhimurium infection are significantly enriched for IL-10-activated genes (*p* = 7.5e−52; 3.53-fold enrichment). In contrast, no enrichment is noted for IL-6-activated genes (*p* = 1) (Figure 1B). Canonical anti-inflammatory targets like *TNIP3,* which is a negative regulator of nuclear factor-κB signaling, are upregulated in both an SarA- and IL-10-dependent manner. *SOCS3* and *SBNO2*, which are known negative regulators of the IL-6 signaling pathway, are significantly upregulated in all three conditions, but are induced to a greater extent during IL-10 stimulation as compared to IL-6 (Figure S1). Thus, there are both qualitative and quantitative differences in STAT3 targets, depending on the stimulus, and the greater overlap of SarA and IL-10 transcriptional targets demonstrates that SarA-dependent activation of STAT3 has an anti-inflammatory bias.

To confirm this SarA-dependent induction of anti-inflammatory targets during infection, we measured the expression levels of anti-inflammatory targets identified from our comparative transcriptomics analysis during wild-type or *ΔsarA Salmonella* Typhimurium infection (Figure 1C). In THP-1 monocytes, wild-type infection leads to significantly higher transcript levels of *SBNO2* and *TNIP3* compared to *ΔsarA.* The induction of these anti-inflammatory genes is not simply secondary to IL-10 production, as THP-1 monocytes demonstrated minimal phospho-STAT3 in response to IL-10 stimulation (Figure S2A). To determine whether differences in bacterial burden could be contributing to the differences in the induction of downstream targets, we carried out a flow cytometric gentamicin protection assay to measure bacterial burden at 3.5 and 8 hours post-infection (hpi). The *ΔsarA* mutant strain had a moderately higher percentage of infected cells and no difference in bacterial replication within THP-1 cells (Figure 1D). Thus, the lower induction of these target genes by *ΔsarA* is not secondary to reduced bacterial burden.

Our data demonstrate that SarA secretion by *Salmonella* Typhimurium during infection promotes an anti-inflammatory response. However, the molecular determinants for this anti-inflammatory bias of SarA are unknown.

### SarA induces more robust and prolonged phosphorylation of STAT3 than gp130 activation

It has previously been demonstrated that the anti-inflammatory bias of IL-10 activation of STAT3 is due to its prolonged and robust phosphorylation of STAT3.^12^ IL-6 leads to a shorter burst of STAT3 phosphorylation compared to IL-10. However, IL-6 signaling can be altered to be anti-inflammatory by prolonging STAT3 activation through the deletion of negative regulators such as SOCS3 or use of constitutively active STAT3 or gp130.^11^^,^^13^ Therefore, we tested whether SarA activation of STAT3 results in more robust STAT3 activation compared to IL-6/gp130 activation.

We previously showed that SarA leads to the activation of STAT3 through a 39-amino acid region of homology with the intracellular portion of the cytokine co-receptor gp130, referred to as the GBS (gp130 binding of STAT3) domain (Figure 1A).^6^ SarA mimics gp130, using a phosphorylated YxxQ motif in the GBS to bind to the STAT3 SH2 domain.^6^ Upon recruitment to the GSK-3-SarA complex, the kinase GSK-3 phosphorylates STAT3 at tyrosine residue 705 (Y705), thereby activating STAT3.^7^ We confirmed that the GBS domains of SarA and constitutively active gp130^15^ are functionally interchangeable, as demonstrated in a time course measuring the ratio of phosphorylated-STAT3-Y705 to total STAT3 protein levels in HeLa cells after overexpressing FLAG-tagged chimeric constructs where the GBS domain of SarA and gp130 had been swapped (Figures 1E and 1F). Levels of Y705 phosphorylation were measured relative to total STAT3 using quantification of infrared fluorescence.^16^^,^^17^ The signal from phospho-STAT3/total STAT3 was used for assessing the relative levels of STAT3 phosphorylation with the overexpression of SarA, gp130, and chimeric constructs but does not represent a measure of the absolute level of STAT3 phosphorylation. Detected phospho-STAT3, STAT3, and β-tubulin were all in the linear range of the assay (Figure S3). While overexpression of all constructs led to STAT3 phosphorylation, constructs containing the SarA GBS domain triggered greater phosphorylation than constructs containing the gp130 GBS at both time points tested. Probing for FLAG showed greater levels of constitutively active gp130 than SarA and that the chimeric proteins are expressed at similar levels to their wild-type counterparts, so decreased STAT3 phosphorylation is not caused by reduced expression (Figure 1E).

We also quantified the abundance of SOCS3 protein, a known anti-inflammatory target of the STAT3 pathway and often used as a measure of STAT3 activation and bias.^10^^,^^13^^,^^18^ Overexpression of constructs containing the SarA GBS domain led to more SOCS3 protein compared to constructs containing the gp130 GBS domain, demonstrating the same pattern as STAT3 phosphorylation (Figure 1G). Thus, overexpression experiments indicate that the SarA GBS causes greater levels of STAT3 activation than the homologous region from gp130 based on both STAT3 phosphorylation and levels of SOCS3 induction.

Similarly, we observed greater STAT3 phosphorylation when comparing *Salmonella* Typhimurium infection with IL-6 stimulation (which signals through gp130 [*IL6ST*] and IL6R heterodimer) in THP-1 monocytes. IL-6 stimulation resulted in an early peak at 30 min. In contrast, SarA activation of STAT3 during infection led to high levels of phosphorylation by 3.5 hpi that were maintained through 24 hpi (Figures 1H and 1I). During infection with a mutant *Salmonella* Typhimurium strain where the GBS of SarA was replaced with the GBS from gp130 (p*sarA:gp130*), the level of phosphorylation never reached the level seen with wild-type SarA and was more similar to the level seen with IL-6 stimulation (Figures 1H and 1I). We also compared phosphorylation levels after infection with the *sarA:gp130*-complemented strain to a *ΔsarA* strain carrying the empty plasmid vector; our results show that while infection with the *ΔsarA* strain does lead to increased STAT3 phosphorylation at 24 hpi, there is still significantly more phosphorylation after infection with the chimeric-complemented strain at 24 hpi (Figure S2B). Infection with p*sarA:gp130* also led to significantly lower SOCS3 abundance compared to p*sarA* (Figure 1J). These differences in STAT3 activation are not due to differences in intracellular bacterial replication (Figure S4). Although we cannot rule out that there may be differences in the level of secretion of the proteins into the host cell, most signals for translocation of T3SS effectors are located in the N-terminal ∼100 amino acids,^19^ and this sequence is shared between SarA and SarA:gp130. Furthermore, the infection data are consistent with the overexpression results, demonstrating that STAT3 activation is reduced when the GBS of SarA is replaced by the GBS of gp130 based on levels of phospho-STAT3 and SOCS3.

Next, we wanted to identify the key molecular differences between the gp130 and SarA GBS domains that are responsible for the difference in robustness of STAT3 activation.

### The greater phosphorylation of STAT3 induced by SarA is not due to a lack of SOCS3 binding

Differences between the GBS domains of gp130 and SarA provided an opportunity to determine the molecular mechanism responsible for the supraphysiological STAT3 activation by SarA. We looked at the role of negative regulators SOCS3 and SHP-2 (encoded by the gene *PTPN11*) on SarA activation of STAT3. Within the GBS domain of gp130, there is a YxxV SHP-2/SOCS3 binding motif. SHP-2 and SOCS3 are the major negative regulators of the canonical gp130/STAT3 pathway. When they bind to this motif on gp130, they may prevent STAT3 binding to the receptor or promote STAT3 degradation; the mechanism is still unclear.^18^^,^^20^^,^^21^^,^^22^ However, SarA does not have the YxxV motif in its GBS domain, suggesting that neither of these negative regulators can bind and downregulate SarA-induced STAT3 phosphorylation.

To test this hypothesis, we measured the effects of RNAi knockdown of SOCS3 and SHP-2 in HeLa cells, which we have previously shown undergoes SarA-dependent STAT3 activation while being easily transfected with robust knockdown.^5^^,^^6^ Following validated knockdown of either SOCS3 or SHP-2 (Figure S5A), cells were treated with oncostatin M (OSM), an IL-6 family cytokine that can signal through OSM receptor (OSMR)-gp130 heterodimer, or infected with either a p*sarA* or p*sarA:gp130* strain of *S.* Typhimurium for 24 h. HeLa cells express higher levels of OSMR than IL-6R,^23^ and we have previously demonstrated that OSM stimulation causes STAT3 phosphorylation in this cell type.^6^ Therefore, we used OSM as a positive control stimulation in this experiment. As with THP-1s, we used flow cytometry to measure the percentage of cells infected and see that HeLa cells are infected to similar levels as THP-1s and to comparable levels for both *Salmonella* strains (Figure S5B, compare with Figure S4).

**Figure 2:**
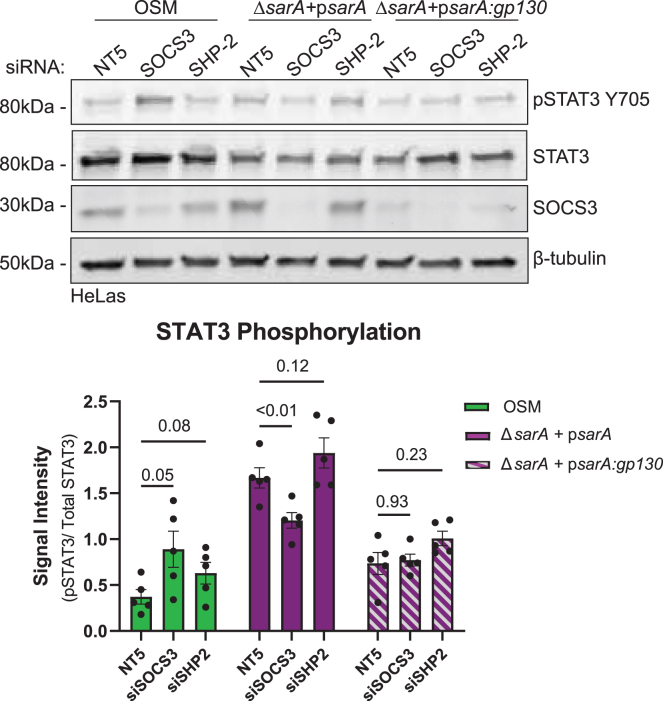
Lack of SOCS3/SHP-2 binding does not explain supraphysiological activation of STAT3 by SarA Knockdown of SOCS3 and SHP-2 do not significantly increase SarA-induced STAT3 phosphorylation. HeLa cells were treated with either a non-targeting control (siGenome non-targeting #5), siSOCS3, or siSHP-2 48 h before stimulation with 10 ng/mL of human OSM or infection with either a wild-type-complemented or psarA:gp130-complemented strain of *Salmonella* Typhimurium. Cell lysates were collected at 24 hpi. STAT3 phosphorylation measured by western blot shows that knocking down negative regulators SOCS3 and SHP-2 was not able to restore psarA:gp130-induced activation to wild-type levels. Data are from five experiments and represented as mean ± SEM. *p* values obtained from a two-way ANOVA with a Dunnett’s multiple comparisons test. See also Figure S5.

As predicted, OSM-dependent STAT3 phosphorylation was increased when SOCS3 was depleted (Figure 2). In contrast, the results show that knocking down SOCS3 or SHP-2 did not increase phospho-STAT3 levels during either p*sarA* or p*sarA:gp130* infection. In fact, through an unknown mechanism, there was a moderate but significant decrease in phospho-STAT3 with *SOCS3* knockdown during p*sarA* infection (Figure 2). Regardless, loss-of-function infection experiments indicate that the loss of SOCS/SHP-2 binding cannot explain the SarA GBS domain’s increased induction of STAT3 phosphorylation compared to the gp130 GBS domain.

### A single amino acid difference controls the robustness of SarA-STAT3 activation

Previously, we determined that a peptide with the phosphorylated YxxQ STAT3 binding motif in SarA binds to STAT3 approximately 16 times stronger than the phosphorylated peptide from gp130, while non-phosphorylated peptides demonstrated no binding to STAT3.^6^ This led us to a second hypothesis for how SarA could lead to greater activation of STAT3: amino acid differences near the YxxQ motif might facilitate greater binding affinity, leading to greater phosphorylation of STAT3.

**Figure 3:**
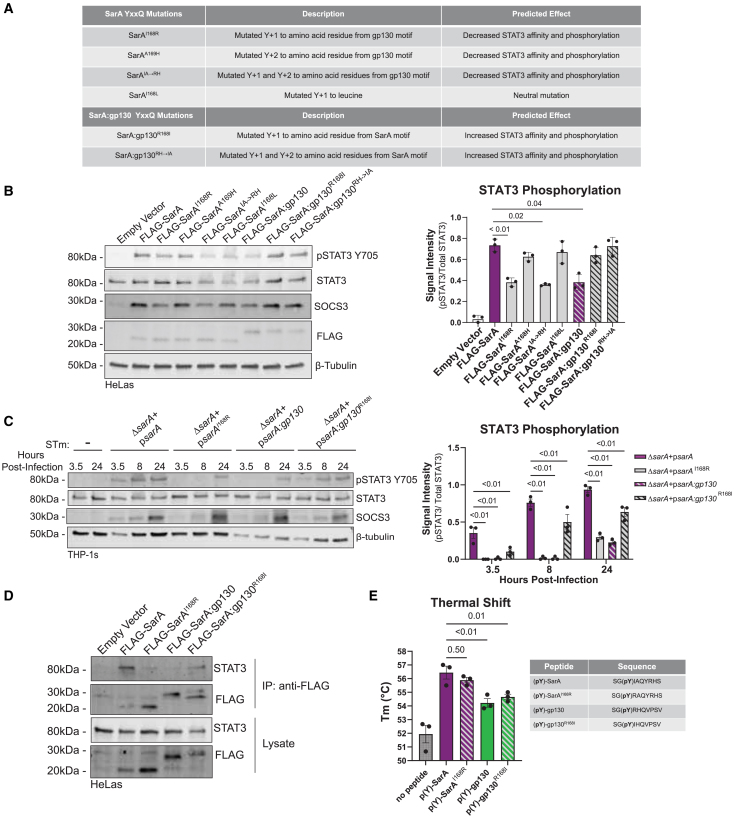
Isoleucine at pY+1 position leads to increased STAT3 binding and phosphorylation (A) Table of mutations made to YxxQ STAT3 binding motif in FLAG-SarA and FLAG-SarA:gp130 constructs. (B) Effects of mutants on STAT3 phosphorylation. Constructs in (A) were overexpressed in HeLa cells. STAT3 phosphorylation measured 24 h post-transfection by western blot shows that mutating the pY+1 position in SarA from isoleucine to arginine leads to a significant decrease in STAT3 activation. Mutating the same position in SarA:gp130 from arginine to isoleucine restores STAT3 phosphorylation to wild-type levels. Mutating the pY+2 position has no significant effect. Data are from three experiments and represented as mean ± SEM. *p* values are from a one-way ANOVA with Dunnett’s multiple comparisons tests comparing all constructs to FLAG-SarA. (C) Isoleucine at the pY+1 position leads to greater STAT3 phosphorylation during infection. THP-1 cells were infected with wild-type-, p*sarA*^I168R^-, p*sarA:gp130*-, or p*sarA:gp130*^R168I^-complemented *Salmonella* Typhimurium. STAT3 phosphorylation was measured by western blot. Data are from three experiments and represented as mean ± SEM. *p* values are from a two-way ANOVA with Tukey’s multiple comparisons test. See also Figure S6. (D) FLAG-SarA constructs with arginine at pY+1 position bind less STAT3. FLAG-SarA, FLAG-SarA^I168R^, FLAG-SarA:gp130, and FLAG-SarA:gp130^R168I^ were overexpressed in HeLa cells for 24 h, followed by co-immunoprecipitation (coIP) and probing for bound STAT3 via western blot. Data are representative of three experiments. (E) Mutating the pY+1 position has a minimal effect on STAT3 binding. Purified STAT3 was incubated with 25 μM phosphopeptides, and a thermal shift assay was used to calculate the melting temperature of the bound peptides as a proxy for binding affinity. Mutating the pY+1 position did not significantly affect melting temperature. Data are from three independent experiments (three technical replicates per experiment were averaged together) and represented as mean ± SEM. *p* values are from Brown-Forsythe and Welch ANOVAs tests with Dunnett’s T3 multiple comparisons test.

Using homology modeling of STAT3 bound to either SarA or gp130 5mer peptides, we predicted which amino acids in the YxxQ STAT3 binding motif might have the greatest effect on binding affinity. The arginine in the pY+1 position of gp130 (R768) appeared to sterically clash with STAT3 at S54. In contrast, the isoleucine in the pY+1 position of SarA (I168) was modeled to form a hydrogen bond with the same S54 residue. Therefore, we generated six FLAG-tagged SarA constructs with mutations in the YxxQ motif (Figure 3A) and overexpressed these constructs in HeLa cells for 24 h. Mutating the pY+1 position of wild-type SarA from isoleucine to arginine (I168R) significantly decreased phospho-STAT3 levels, down to the level of SarA:gp130. In contrast, mutating the pY+1 position in SarA:gp130 from arginine to isoleucine (R168I) significantly increased phospho-STAT3 to wild-type levels (Figure 3B). Notably, switching the pY+2 position or making a conservative pY+1 change (isoleucine to leucine) had a minimal effect on phospho-STAT3 levels.

To confirm this result during *Salmonella* Typhimurium infection, we generated strains of *Salmonella* Typhimurium that express SarA with the same YxxQ mutations. We infected THP-1 cells with these strains and measured phospho-STAT3 levels over the course of a 24-h infection. Our results show that the p*sarA*^I168R^ mutant strain has significantly slower and lower overall amounts of STAT3 phosphorylation during infection compared to the wild-type complemented strain; this phenocopies the pattern of phosphorylation during infection with the p*sarA:gp130*-expressing strain (Figure 3C). During infection with the p*sarA:gp130*^R168I^ mutant strain, STAT3 phosphorylation was significantly increased compared to infection with the p*sarA:gp130* strain (Figure 3C). We again confirmed that these differences in STAT3 activation are not due to differences in intracellular bacterial burden (Figure S6).

To determine whether the pY+1 mutations affect STAT3 phosphorylation due to changes in STAT3 binding, we performed co-immunoprecipitation (coIP) experiments using our FLAG-tagged SarA overexpression constructs. The SarA^I168R^ mutant exhibited reduced binding to STAT3, down to the level seen with the SarA:gp130 chimera (Figure 3D). In contrast, the SarA:gp130^R168I^ mutant had levels of STAT3 binding that were similar to the wild-type SarA construct (Figure 3D).

We then tested whether the pY+1 mutations changed the binding affinity to STAT3 as the differences in phosphorylation levels and binding of STAT3 in cells would suggest. Previously, thermal shift assays (TSAs) were used to demonstrate binding of gp130 peptide to STAT3.^24^ Therefore, we developed a TSA to measure the thermostability of recombinant, purified STAT3 in the presence of phosphopeptides derived from the SarA and gp130 STAT3 binding sites. The TSA served as a proxy for direct measurement of binding affinity. The TSA recapitulated the greater affinity of STAT3 for the phospho-SarA peptide compared to the phospho-gp130 peptide. Surprisingly, mutating the peptides at the pY+1 position resulted in no significant difference in binding affinity (Figure 3E), indicating that enhanced binding affinity was not the primary mechanism by which the I168R and R168I mutations were exerting their effect on STAT3 activation.

### Isoleucine at the pY+1 position makes SarA a better substrate for GSK-3 tyrosine phosphorylation

**Figure 4:**
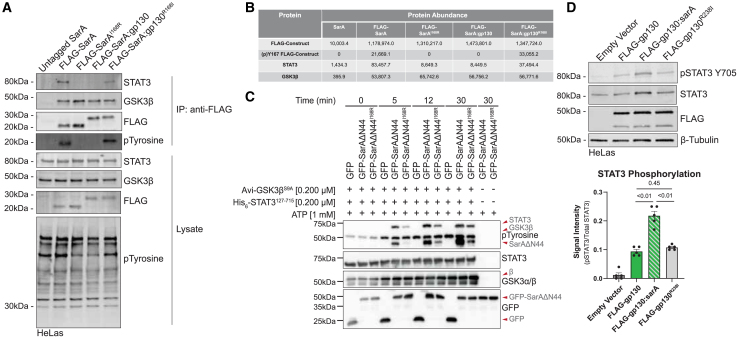
Isoleucine at the pY+1 position makes SarA a better substrate for GSK-3, but it has no effect on gp130 (A) Isoleucine at the pY+1 position is required for SarA phosphorylation. Empty vector and panel of FLAG-SarA constructs were overexpressed in HeLa cells, and coIP confirms that while GSK-3 is bound to all constructs, tyrosine phosphorylation of SarA^I168R^ and SarA:gp130 was not detected. Data are representative of three experiments. (B) IP-MS results show how pY+1 mutation affects abundance of proteins bound to each SarA construct. Data from one IP-MS experiment confirming IP-western results in (A). See also Figure S7. (C) Mutation of isoleucine at the pY+1 position greatly reduces phosphorylation of SarA by GSK-3. GFP, GFP-SarAΔN44, and GFP-SarAΔN44^I168R^ were expressed in *GSK-3α/β*^−/−^ 293ET cells, immunoprecipitated, and assessed for their ability to be tyrosine phosphorylated by GSK-3β in an *in vitro* kinase assay containing 1 mM ATP with or without recombinant Avi-GSK-3βS9A (0.2 μM) and His6-STAT3127-715 (0.2 μM). Data are representative of three experiments. (D) Isoleucine at the pY+1 position is not sufficient to induce greater levels of gp130-mediated STAT3 phosphorylation. Empty vector, FLAG-gp130, FLAG-gp130:sarA, and FLAG-gp130^R238I^ were overexpressed in HeLa cells for 24 h, STAT3 phosphorylation assessed by western blot shows that the R238I single-point mutation does not increase pSTAT3 levels compared to wild-type gp130. Data are from four experiments and represented as mean ± SEM. *p* values obtained from Brown-Forsythe and Welch ANOVAs with Dunnett’s T3 multiple comparisons test.

As binding of SarA to STAT3 requires phosphorylation of the tyrosine in the YxxQ motif,^6^ we hypothesized that the reduced binding of STAT3 could be secondary to the level of SarA phosphorylation. Immunoprecipitates of FLAG-SarA demonstrated that while the constructs bound similar levels of GSK-3β, there was much greater tyrosine phosphorylation in the molecular weight ranges of SarA and gp130:SarA (22–28 kDa) for the constructs with isoleucine, but not arginine, at the Y+1 position (Figure 4A). Both SarA and SarA:gp130 have multiple isoforms, presumably due to post-translational modification as previously reported.^6^^,^^7^ The critical importance of isoleucine at the Y+1 position for SarA phosphorylation was further validated by liquid chromatography-tandem mass spectrometry (LC-MS/MS) analysis of immunoprecipitates (Figure 4B; Table S1). While non-phosphorylated forms of all proteins were detected by LC-MS/MS, Y167 phosphorylation was only measured on wild-type FLAG-SarA and FLAG-SarA:gp130^R168I^ but not the constructs with arginine in the pY+1 position (Figures 4B and S7). The LC-MS/MS analysis further confirmed that the amount of STAT3 co-immunoprecipitated was greater for the constructs with isoleucine at the Y+1 position, but binding of GSK-3β to FLAG-SarA or FLAG-gp130:SarA was not influenced by amino acids in this position (Figure 4B). Collectively, these data suggest that an isoleucine in the pY+1 of the YxxQ (i.e., YIxQ) is critical for the phosphorylation of SarA or SarA:gp130 and subsequent STAT3 binding.

This increased cellular phosphorylation of Y167 is evidence that isoleucine at the pY+1 position makes the YxxQ motif a better substrate for GSK-3 tyrosine phosphorylation. Previously, the Thurston lab demonstrated the direct phosphorylation of SarA by GSK-3, revealing the first exogenous substrate of GSK-3 tyrosine phosphorylation.^7^ While GSK-3 is canonically a serine-threonine kinase, it does autophosphorylate its activation loop at Y216 in GSK-3β (phosphorylation in the GSK-3 activation loop was also confirmed by LC-MS/MS in all SarA and SarA:gp130 immunoprecipitates).^25^ Therefore, to measure the effect of the Y+1 isoleucine on direct GSK-3 phosphorylation of SarA, we conducted an *in vitro* kinase assay using SarA containing isoleucine or arginine at the pY+1 position as well as recombinant STAT3 and GSK-3β. We observed greater tyrosine phosphorylation of SarA by GSK-3β with isoleucine present at the pY+1 position at all time points tested, and this corresponded with greater tyrosine phosphorylation of STAT3 (Figure 4C). The sequence encoding the N-terminal 44-amino acid residues of SarA was deleted, as these residues do not appear to affect activity, and this removes an additional tyrosine phosphorylation site to simplify interpretation. Thus, isoleucine at pY+1 renders SarA a better substrate for GSK-3 tyrosine phosphorylation.

### Constitutively active gp130 does not induce greater STAT3 activation with isoleucine at the pY+1 position

The altered levels of STAT3 phosphorylation induced with the chimeric SarA constructs depend on the presence of GSK-3 as the cognate kinase. Therefore, it was unclear whether isoleucine at the pY+1 position would have a similar effect within gp130, which uses Janus kinases (JAKs) instead of GSK-3. To test this, we made an R238I mutation in the constitutively active gp130 dimer construct. While replacing the entire 39-amino acid GBS segment with the homologous SarA sequence resulted in greater phospho-STAT3 levels (consistent with our previous studies^6^), overexpression of the R238I mutant in HeLa cells did not alter phopho-STAT3 levels compared to the wild-type gp130 construct (Figure 4D).

### Tuning SarA activation of STAT3 controls the bias of target genes

**Figure 5:**
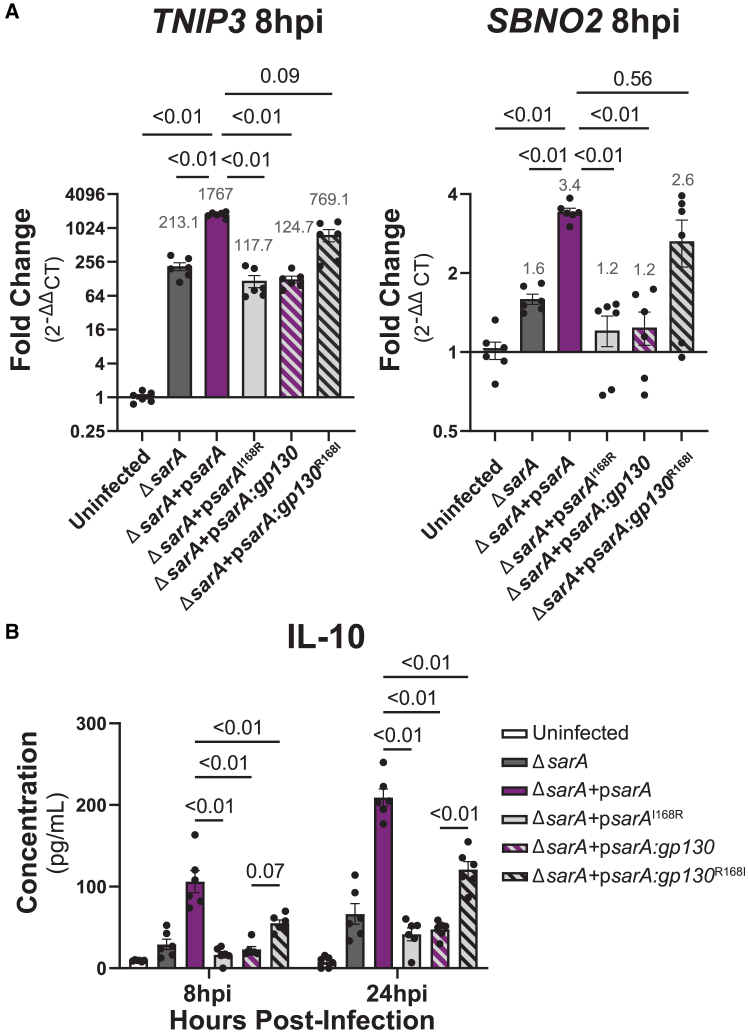
Isoleucine at pY+1 position promotes anti-inflammatory signaling bias (A) Arginine in the pY+1 position leads to decreased expression of anti-inflammatory genes during infection, and isoleucine at this position rescues the expression of these genes. THP-1 cells were infected with *ΔsarA*, wild-type-complemented, p*sarA*^I168R^, p*sarA:gp130*, or p*sarA:gp130*^R168I^*Salmonella* Typhimurium. RNA was collected from cells 8 hpi and expression levels of *SBNO2* and *TNIP3* were measured via qPCR. Data points are from six biological replicates across three experiments (with each biological replicate shown being the average of three qPCR technical replicates). Gray values above bars are the mean. Data are represented as mean ± SEM. *p* values obtained from Brown-Forsythe and Welch ANOVAs with Dunnett’s T3 multiple comparisons test. (B) Arginine in the pY+1 position leads to decreased production of IL-10 during infection and is rescued with isoleucine at the position. THP-1 cells were infected with *ΔsarA*, wild-type-complemented, p*sarA*^I168R^, p*sarA:gp130*, or p*sarA:gp130*^R168I^*Salmonella* Typhimurium. Cell supernatant was collected at 8 and 24 hpi, and IL-10 was measured via ELISA. Data are from six biological replicates across three experiments and represented as mean ± SEM. *p* values obtained from a two-way ANOVA with Sidak’s multiple comparisons test.

Does supraphysiological activation of STAT3 impact downstream transcriptional targets? To test this, we infected THP-1s with the YxxQ mutant strains of *Salmonella* Typhimurium and measured the transcription and production of downstream anti-inflammatory STAT3 targets. Our results demonstrate that the p*sarA*^I168R^ mutation leads to reduced transcription of anti-inflammatory genes down to p*sarA:gp130* levels, and increased transcription is restored with the p*sarA:gp130*^R168I^ mutation (Figure 5A). Infection with the p*sarA*^I168R^ mutant strain also leads to significantly lower production of IL-10 compared to wild type, whereas infection with the p*sarA:gp130*^R168I^ mutant leads to significantly increased IL-10 compared to the p*sarA:gp130* strain (Figure 5B).

### Evolution of STAT3 activation by SarA vs. gp130

**Figure 6:**
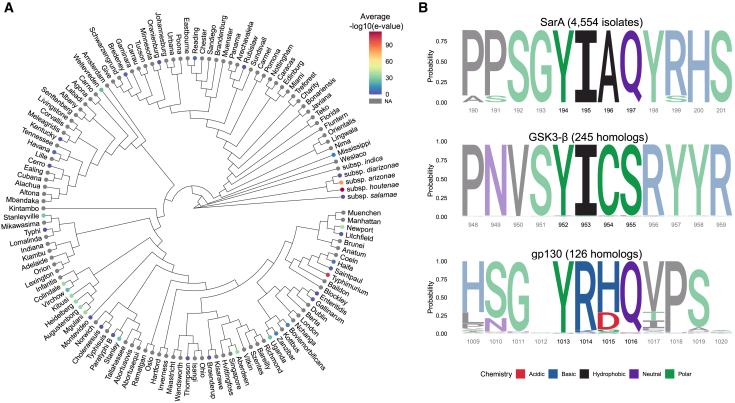
SarA is variably present in *Salmonella* serovars, but isoleucine at pY+1 position is always conserved (A) SarA is present in diverse *S. enterica* subspecies and serovars. Mean E-values (indicated by color) from BLASTp of SarA in 21,223 *Salmonella* isolates were merged with an existing whole-genome maximum-likelihood phylogeny.^27^ Serovars without corresponding BLASTp data are in gray. (B) Isoleucine at pY+1 is invariable across *Salmonella* isolates that contain SarA and homologs of the human protein GSK-3β. Sequence logos were derived from multiple sequence alignments and reflect the relative frequency of each residue across all aligned sequences. The YxxQ motif that is homologous between SarA and IL6ST is highlighted, as is the known autophosphorylation site in the kinase GSK-3β. Known chemical classification of each residue is indicated by color.

Our results show that the amino acid in the pY+1 position of the YxxQ motif in SarA leads to increased phosphorylation by GSK-3, which causes increased STAT3 binding and increased phospho-STAT3 levels compared to the residue in the same position in gp130. SarA is present on the Gifsy-1 prophage^5^ and can therefore be acquired through horizontal gene transfer among serovars. BLASTp of SarA in over 20,000 *Salmonella* strains^26^ demonstrated the presence of SarA in 4,554 strains, including members of the Arizonae and Houtenae subspecies that primarily infect birds and reptiles (Figure 6A). In these 4,554 isolates, the pY+1 position is invariably isoleucine (Figure 6B). Thus, all examined *Salmonella* strains that encode for SarA have retained the isoleucine at the pY+1 position that makes SarA’s YxxQ motif a better substrate for GSK-3, facilitating supraphysiological activation of STAT3.

Isoleucine at this pY+1 position also shows conservation at the autophosphorylation site of GSK-3β across 244 homologs of human GSK-3β in 197 species extending back to yeast (Figure 6B; Table S2). Thus, maintaining high activity of this autophosphorylation event appears to be under evolutionary constraint, consistent with GSK-3’s high baseline activity in unstimulated cells.^28^ In contrast, multiple sequence alignment comparison of 133 homologs of human gp130 showed that the pY+1 position is almost invariably arginine across 131 species extending back to fish (Figure 6B). The exceptions are changes to the similarly basic lysine residue in spotted gar, elephant shark, Old Calabar mormyrid elephantfish, and Asian bonytongue fish. This indicates conservation at the pY+1 position extending to the common ancestor of fish and tetrapods in the Devonian period ∼400 million years ago.^29^ While R238I is not sufficient to increase the activity of gp130 (see Figure 4C), greater activation of STAT3 by gp130 is possible with further mutagenesis of the GBS based on the gp130-SarA chimeric construct. Therefore, gp130 appears to have evolved to mediate an intermediate level of activation of STAT3.

## Discussion

Many intracellular pathogens, including both bacteria and viruses, have been shown to activate anti-inflammatory pathways during infection to evade host pro-inflammatory attacks.^30^^,^^31^ In this paper, we demonstrate that a single amino acid residue in the YxxQ motif of *Salmonella* Typhimurium effector SarA controls the level of STAT3 activation to induce IL-10 production and cause an anti-inflammatory signaling bias in infected cells. SarA signaling through STAT3 drives M2 macrophage polarization during systemic *Salmonella* Typhimurium infection,^7^^,^^8^^,^^9^ while IL-10 secreted by T and B cells is required for dissemination of *Salmonella* Typhimurium from the gut to systemic sites of infection in a mouse model.^32^ SarA-induced IL-10 production could lead to non-cell-autonomous responses that benefit the bacteria, such as decreasing production of pro-inflammatory cytokines by bystander cells, preventing the recruitment of other immune cell populations, and reducing T cell responses. The relative contributions of these possible effects of SarA during *Salmonella* infection are an important direction for future work.

Beyond *Salmonella,* other bacteria also hijack STAT3 through different mechanisms. *Bartonella hensalae*, which causes cat scratch disease in humans, employs the effector BepD to activate STAT3 in a c-Abl kinase-dependent mechanism to suppress tumor necrosis factor-α signaling and stimulate IL-10 production.^33^ While BepB signaling in this context has an anti-inflammatory bias similar to SarA activation, it uses a kinase that, unlike GSK-3, is not active at baseline.^34^ *Helicobacter pylori*, which typically causes chronic infection of the stomach, also manipulates STAT3 signaling. Its effector CagA is notable for being able to either activate or suppress IL-6/gp130 signaling depending on the effector’s phosphorylation state.^35^ CagA has multiple phosphorylation sites that have been shown to be phosphorylated by c-Src and c-Abl kinase families,^36^ suggesting that the effector’s signaling pathway can be tightly controlled, and different combinations of phosphorylation may occur in dynamic *H. pylori* populations during infection. Furthermore, as CagA acts through IL-6 and gp130 signaling for STAT3 activation, the features of this host signaling pathway that reduce STAT3 signaling (JAK dependence, inhibition by SOCS proteins) are still at play in this context. Thus, while multiple pathogens can activate STAT3, the magnitude of STAT3 activation may be adapted to each particular pathogen. Whereas supraphysiological activation of STAT3 with strong anti-inflammatory bias may be adaptive for SarA-containing *Salmonella* serovars that typically cause acute gastroenteritis in human hosts, more nuanced control may be utilized by *H. pylori* in causing chronic infection. The variable presence of SarA among *Salmonella* serovars suggests complex bacterial adaptation to different host species and requires further investigation into how triggering supraphysiological activation of STAT3 affects various pathologies.

Effector activation of STAT3 may at first seem counterintuitive, as the host is already producing IL-6 in response to *Salmonella* infection,^37^^,^^38^^,^^39^ and we previously observed activation of STAT3 in infected mice even in the absence of SarA.^5^ SarA appears to exploit a homeostatic mechanism, whereby the host uses the same transcription factor (STAT3) to induce pro-inflammatory, and at greater levels of activation, anti-inflammatory targets. Short, rapid phosphorylation of STAT3 via IL-6 signaling leads to pro-inflammatory target genes being activated. In contrast, continued and more robust activation of this same transcription factor via IL-10 signaling leads to the activation of anti-inflammatory target genes.^14^ Understanding how a limited set of shared proteins mediate the very different effects of IL6, IL-10, and other cytokines that induce JAK/STAT signaling is still a very active area of research.^40^ One mechanism underlying this difference between IL-6 and IL-10 signaling is responsiveness to negative regulators: SHP-2 and SOCS3 bind to gp130 to inhibit signaling, but neither of these proteins can downregulate IL-10 signaling.^41^ It has also been shown that deletion of SOCS3 or mutation of its binding site in gp130 results in IL-6 displaying an anti-inflammatory bias similar to IL-10 signaling.^11^^,^^18^^,^^42^

We initially hypothesized that SarA was able to manipulate STAT3 phosphorylation dynamics by avoiding these negative regulators, similar to IL-10. Instead, we found that *Salmonella*-mediated activation of STAT3, by co-opting the constitutively active kinase GSK-3, is what determines the downstream signaling bias. Both IL-6 and IL-10 signaling utilize strict control of the JAK kinases that activate STAT3, forming an active complex with JAK and STAT3 upon cytokine stimulation. GSK-3 is a serine/threonine kinase with over 100 known substrates and plays an important role in metabolic regulation, proliferation, and inflammation.^43^^,^^44^ Despite exhibiting strict serine/threonine activity in its mature state, GSK-3 is sometimes classified as a dual-specificity kinase, as it autophosphorylates a tyrosine residue in its activation loop during translation.^25^ Prior to the discovery of SarA and STAT3 phosphorylation by GSK-3,^7^ autophosphorylation at the YICS sequence in the GSK-3 activation loop, which is required for full protein activity, was the only known substrate for this activity.^25^ Our results show that SarA has evolved to match the GSK-3 autophosphorylation site to facilitate constitutive STAT3 activation in a cytokine-independent manner. Notably, the phosphorylation site in STAT3 targeted by GSK-3 has leucine at the pY+1 position (L706), a conservative change that at least in the SarA YxxQ motif has no effect on phosphorylation (see Figure 3B). The autophosphorylation of GSK-3 occurs primarily during the folding process, in a chaperone-dependent manner.^25^ How SarA is able to re-awaken this activity is unknown, but in addition to having the optimal substrate sequence with isoleucine in the pY+1 position, there may be structural contributors to this. The GBS region in SarA is rich in prolines and serines (12 of 39 residues), which we speculate may result in an extended, poorly structured sequence. While our work reveals a molecular basis as to how SarA is able to activate STAT3 to supraphysiological levels, future work will be needed to define the structural basis that underlies these findings and whether there are more substrates for the tyrosine kinase activity of GSK-3.

### Limitations of the study

This work must be interpreted in light of limitations of study design and experimental systems used. Our experiments were focused on isolating the function of the shared GBS regions of SarA and gp130, so the importance of other regions of SarA were not interrogated in this work and may impact the functions studied in this manuscript. Our experiments primarily used overexpression in HeLa cells and infection assays in THP-1 monocytes, so other cell types may demonstrate differences in SarA function. Experiments in this study used cell lines and purified binding and kinase assays but did not include animal or organoid models necessary to draw further conclusions about the physiological consequences of supraphysiological activation of STAT3 by SarA.

## Resource availability

### Lead contact

Further information and requests for resources and reagents should be directed to and will be fulfilled by the lead contact, Dennis C. Ko (dennis.ko@duke.edu).

### Materials availability

Plasmids generated by this study are being deposited to AddGene. Bacterial strains generated by this study are available upon request.

### Data and code availability

• Raw and processed MS data and associated metadata have been deposited to the ProteomeXchange Consortium PXD056916 via the MassIVE repository and can be accessed at MassIVE repository: ftp://massive.ucsd.edu/v08/MSV000096120/.
• All original code has been uploaded to GitHub: https://github.com/Angela-Jones/salmonella_sarA and is publicly available as of the date of publication.
• Any additional information required to reanalyze the data reported in this paper is available from the lead contact upon request.

## Acknowledgments

We thank the members of the Ko lab for useful discussion. We thank the Duke University School of Medicine for the use of the Proteomics and Metabolomics Core Facility, which provided proteomics services. M.R.G. was supported by 10.13039/100000002NIH 1F31AI176719-01A1. D.C.K., A.G.J., and R.E.L. were supported by 10.13039/100000002NIH R01AI118903. T.L.M.T. was supported by the 10.13039/501100000268Biotechnology and Biological Sciences Research Council David Phillips Fellowship (BB/R011834/1), 10.13039/501100000265Medical Research Council research grant no. MR/V031058/1 (which also funded J.H.M.), and a 10.13039/501100000781European Research Council grant funded by the 10.13039/501100000266Engineering and Physical Sciences Research Council EP/X02377X/1. (which also funded I.P.). E.J.W was supported by the Duke Next Generation Fellowship funded by the Duke Science and Technology Institute. All schematic images were generated using Biorender.com, and figures were made with Adobe Illustrator.

## Author contributions

Conceptualization, M.R.G. and D.C.K.; formal analysis, M.R.G., A.G.J., and M.W.F.; investigation, M.R.G., A.G.J., I.P., M.W.F., E.J.W., R.E.L., and D.C.K.; funding acquisition, T.L.M.T. and D.C.K.; supervision, R.G.B., T.L.M.T., and D.C.K.; resources, M.R.G., A.G.J., I.P., R.E.L., J.H.M., E.J.W., R.G.B., T.L.M.T., M.W.F., and D.C.K.; writing – original draft, M.R.G. and D.C.K.; writing – review & editing, M.R.G., A.G.J., R.E.L., I.P., E.J.W., T.L.M.T., M.W.F., and D.C.K.

## Declaration of interests

The authors declare no competing interests.

## STAR★Methods

### Key resources table

**Table:** 

REAGENT or RESOURCE	SOURCE	IDENTIFIER
**Antibodies**		

Anti-FLAG M2	Sigma	Cat#F3165; RRID:AB_259529
Anti-pY705-STAT3 clone D2A7	CST	Cat#9145; RRID:AB_2491009
Anti-STAT3 clone 124H6	CST	Cat#9139; RRID:AB_331757
Anti-SOCS3 polyclonal	Proteintech	Cat#14025-1-AP; RRID:AB_10597854
Anti-GSK3β clone D5C5Z	CST	Cat#12456; RRID:AB_2636978
Anti-pTYR-1000 monoclonal pool	CST	Cat#8954; RRID:AB_2687925

**Bacterial and virus strains**		

*S. enterica* Typhimurium 14028s + p67GFP3.1	Dennis Ko	DCK22
*S. enterica* Typhimurium 14028s Δ*sarA* + p67GFP3.1	Sarah Jaslow^5^	DCK444
*S. enterica* Typhimurium 14028s Δ*sarA* + pWSK129	Sarah Jaslow^5^	DCK486
*S. enterica* Typhimurium 14028s Δ*sarA* + pWSK129-*sarA +* p67GFP3.1	Sarah Jaslow^5^	DCK487b
*S. enterica* Typhimurium 14028s Δ*sarA* + pWSK129-*sarA:gp130* + p67GFP3.1	This study	DCK1156
*S. enterica* Typhimurium 14028s Δ*sarA* + pWSK129-sarA^168R^ + p67GFP3.1	This study	DCK1222
*S. enterica* Typhimurium 14028s Δ*sarA* + pWSK129-sarA:gp130^R168I^ + p67GFP3.1	This study	DCK1223

**Chemicals, peptides, and recombinant proteins**		

OSM	PeproTech	Cat#300-10
IL-6	PeproTech	Cat#200-06
IL-10	PeproTech	Cat#200-10
Gentamicin Sulfate	Sigma	Cat#G3632-5G
7-aminoactinomycin D (7AAD)	Enzo	ALX-380-283
Isopropyl ß-D-1-thiogalactopyranoside (IPTG)	ThermoFisher	Cat#15529–019
Taqman FAM-MGB *TNIP3* probe	ThermoFisher	4448892, Hs00375573
Taqman FAM-MGB *SBNO2* probe	ThermoFisher	4448892, Hs00922127
Taqman FAM-MGB *SOCS3* probe	ThermoFisher	4453320, Hs02330328
Taqman FAM-MGB *PTPN11* probe	ThermoFisher	4453320, Hs00275784
Taqman FAM-MGB RNA18S5 probe	ThermoFisher	4331182, Hs03928985
siGENOME Non-Targeting #5	Dharmacon	Cat#D-001210-05
siGENOME *SOCS3* SMARTpool siRNA	Dharmacon	Cat#M-004299-02-0005
siGENOME *PTPN11* SMARTpool siRNA	Dharmacon	Cat#M-003947-01-0005
25μM gp130 phosphopeptide	Genscript	SGpYRHQVPSV
25μM gp130^R168I^ phosphopeptide	Genscript	SGpYIHQVPSV
25μM SarA phosphopeptide	Genscript	SGpYIAQYRHS
25μM SarA^I168R^ phosphopeptide	Genscript	SGpYRAQYRHS
PTMScan Wild Type Alpha-Lytic Protease (WaLP)	Cell Signaling	Cat#33036
10X Kinase Buffer	Cell Signaling	Cat#9802

**Critical commercial assays**		

Quikchange II XL Site-Directed Mutagenesis Kit	Agilent	Cat#200521
Lipofectamine 3000	ThermoFisher	Cat#L3000008
Lipofectamine RNAiMAX	ThermoFisher	Cat#13778075
Human IL-10 ELISA	R&D Systems	Cat#DY217B
RNeasy kit	Qiagen	Cat#74106
iScript cDNA Synthesis kit	BioRad	Cat#1708891
iTaq Universal Probes Supermix	BioRad	Cat#1725134
1X Glomelt Thermal Shift Protein Stability Kit	Biotium	Cat#33021-1

**Deposited data**		

Dynamic genome-wide transcriptional response of human monocyte-derived dendritic cells to IL-6 and Il-10	Braun et al. 2013, GEO	GSE45466
Mass spectrometry of FLAG-SarA immunoprecipitates	MassIVE repository	MSV000096120
*Salmonella* genomes	PubMLST	www.pubmlst.org

**Experimental models: Cell lines**		

THP-1 Cells	Duke Cell Culture Facility, originally from ATCC	TIB-202
HeLa Cells	Duke Cell Culture Facility, originally from ATCC	CCL-2

**Oligonucleotides**		

See Table S4 for list of primers.	This study	See Table S4

**Recombinant DNA**		

See Table S3 for plasmids.	This study	See Table S3

**Software and algorithms**		

GraphPad Prism 10	GraphPad Software	www.graphpad.com
R 4.3.1	R Core Team	www.r-project.org
BioRender	BioRender	www.biorender.com
Spectronaut 19.2	BIOGNOSYS	www.biognosys.com/resources/spectronaut-19-unlock-your-datas-true-story
*treeio* v.1.26.0	Wang et al. 2020^27^	www.github.com/YuLab-SMU/treeio
*ggseqlogo* v.0.1	Wagih et al. 2017^45^	www.github.com/omarwagi/ggseqlogo

### Experimental model and subject details

#### Plasmids

Plasmids are listed in Table S3. The pcDNA3.1-FLAG*-sarA*, pcDNA3.1-FLAG*-sarA:gp130* chimera, pcDNA3.1-FLAG-*gp130dimer*, pcDNA3.1-FLAG*-gp130:sarA* chimera codon-optimized for mammalian expression were previously described in Gibbs et al., 2020 and are available from Addgene. Site-directed mutagenesis was carried out using Quikchange II XL Site-Directed Mutagenesis Kit (Agilent, #200521) (primers in Table S4). Note that amino acid numbering used throughout this manuscript is based on the Uniprot entry for SarA (A0A0F6B506). Panagi et al. noted that the translational start very likely occurs 72 nucleotides (24 amino acid residues) later than indicated,^7^ but as all of our human codon-optimized SarA plasmids contain these additional bases, we have indicated the critical isoleucine residue as being at amino acid position 168.

#### Mammalian cell culture

THP-1s (Duke Cell Culture Facility, Male) were cultured at 37°C in 5% CO2 in RPMI 1640 media (Invitrogen) supplemented with 10% heat-inactivated FBS (Thermo-Fisher), 2 μM glutamine, 100 U/mL penicillin-G, and 100 mg/mL streptomycin. HeLa cells (Duke Cell Culture Facility, Female) were grown in high glucose DMEM media supplemented with 10% FBS, 1mM glutamine, 100 U/mL penicillin-G, and 100 mg/mL streptomycin. Cells used for *Salmonella* gentamicin protection assays were grown without antibiotics for at least 1 h prior to infection. All cell lines have been tested for mycoplasma contamination.

#### Bacteria

All *Salmonella* strains are derived from the *S*. Typhimurium strain 14028s and are listed in Table S5, and all plasmids are listed in Table S3. For infection of cells, bacteria were grown overnight in LB broth (Miller formulation, BD), subcultured 1:33 in 1mL cultures, and grown for an additional 160 min at 37°C shaking at 250 RPM. Ampicillin was added to LB at 100 μg/mL, kanamycin at 50 μg/mL

### Method details

#### Transfections

HeLa cells were seeded at 1.75 × 10^5^ cells per well in 6-well TC-treated plates or 7.5 × 10^4^ cells in 24-well TC-treated plates 24 h before transfection. Transfection was accomplished with Lipofectamine 3000 (Thermo Fisher, Catalog #L3000008). Cells were harvested 16, 24, and 48 h post transfection.

#### Cellular treatments

Recombinant human cytokines, OSM (PeproTech 300–10), IL-6 (PeproTech 200-06), and IL-10 (PeproTech 200–10), were resuspended in water and incubated with cells for indicated time and concentration.

#### Infection assays

Assaying infection of THP-1s and HeLa cells was done as previously described.^46^ Overnight cultures of *Salmonella* in LB media were subcultured 1:33 and grown for 160 min at 37°C and 250 rpm. THP-1s were seeded at either 100,000 in 100 μL in 96-well or 500,000 in 500ul in 24-well non-TC plates and infected at multiplicity of infection (MOI) 10. HeLa cells at 300,000 in 2 mL were infected at MOI 5 in 6-well TC-treated plates. Gentamicin was added 1 hpi at 50 μg/mL to kill the extracellular bacteria. For 24-h incubations, gentamicin was diluted to 15 μg/mL at 2 hpi. To induce GFP, IPTG was added 75 min prior to the desired timepoint. Infection and cell death were measured with a Guava EasyCyte Plus flow cytometer (Millipore). Cell death was measured by 7AAD (7-aminoactinomycin D; Enzo Life Sciences) staining. IL-10 protein in the THP-1 supernatant at 8 and 24 hpi was measured by human IL-10 ELISA (R&D systems Catalog #DY217B).

#### Measuring anti-inflammatory target gene expression

To measure *TNIP3* and *SBNO2* induction, 5 x 10^5^ THP-1s in a 24-well non-TC treated plate were infected with *S.* Typhimurium at MOI10 as described above or stimulated with 10 ng/mL of IL-6 as described above. At 24hpi, RNA was harvested and *TNIP3* and *SBNO2* expression were measured using qPCR. Briefly, RNA was harvested using a RNeasy kit (Qiagen, #74106), cDNA was generated with iScript synthesis kit (Bio-Rad, #1708891), and qPCR was performed by using iTaq Universal Probes Supermix (Bio-Rad, #1725134) and a QuantStudio 3 thermo cycler (Applied Biosystems). Taqman probes are listed in Key Resources Table. The cycling conditions were as follows: 50°C for 2 min, 95°C for 10 min, and 40 cycles of 95°C for 15 s followed by 60°C for 1 min. All qPCR was run in technical triplicate. The comparative threshold cycle (CT) was used to quantify transcripts, with the ribosomal 18s gene (RNA18S5) serving as the housekeeping control. ΔCT values were calculated by subtracting the CT value of the control gene from the target gene, and the ΔΔCT was calculated by subtracting the non-targeting siRNA ΔCT from the targeting siRNA ΔCT value. Fold change represents 2^−ΔΔCT^.

#### Immunoprecipitation

HeLa cells were seeded at 1.75 × 10^6^ cells per 10 cm dish 24 h before transfection with codon-optimized pcDNA3.1-Flag-sarA, pcDNA3.1-FLAG-sarA:gp130, pcDNA3.1-FLAG-sarA^I168R^, or pcDNA3.1-FLAG-sarA:gp130^R168I^ as described above. Four plates per condition were lysed in 1mL of 0.1% Triton X-100, 50mM Tris pH 7.4, 150mM NaCl, cOmplete Mini protease inhibitor cocktail (Millipore Sigma #11836170001), 10mM NaF, and 1-mM Na Orthovanadate. Lysate was incubated with anti-FLAG M2 magnetic beads (Millipore Sigma #M8823) for 4 h while rotating at 4C then washed 3x with 200μL of lysis buffer using a DynaMag-2 magnet (Invitrogen #12321D). Bound protein was eluted with 45μL of 0.1-M Glycine-HCl buffer pH 3 and neutralized with 5μL of 0.5M Tris-HCl pH 7.4 1.5M NaCl. Eluted proteins were analyzed by immunoblot.

#### Immunoblotting

Cells were lysed with RIPA lysis buffer: 50mM Tris-HCl pH 7.4, 150mM NaCl, 0.1% SDS, 0.5% NaDeoxycholate, 1% Triton X-100, cOmplete Mini protease inhibitor cocktail (Millipore Sigma #11836170001) 10mM NaF, and 1-mM Na orthovanadate. SDS-PAGE was performed using Mini-PROTEAN TGX Stain-Free Precast 4%–20% gels (Bio-Rad #456–8096). The gels’ stain-free dye was activated by a 5-min UV exposure and protein was transferred to Immun-Blot low-fluorescence PVDF membrane (Bio-Rad #162–0264) using Hoefer TE77X. Primary antibodies used were: anti-FLAG M2 (Sigma F3165, RRID:AB_259529), anti-pY705-STAT3 clone D3A7 (CST #9145, RRID:AB_2491009), anti-STAT3 clone 124H6 (CST #9139, RRID:AB_331757), anti-SOCS3 polyclonal (Proteintech #14025-1-AP, RRID:AB_10597854), anti-GSK3β clone D5C5Z (CST #12456, RRID:AB_2636978) and anti-pTyr-1000 monoclonal pool (CST #8954, RRID:AB_2687925. Blots were developed using LI-COR infrared secondary antibodies (IRDye 800CW Donkey anti-rabbit IgG and IRDye 680LT Donkey anti-mouse IgG) and imaged on an LI-COR Odyssey Classic. Total protein was measured following a 30-s UV exposure. Band integrated intensity was quantified using Image Studio v5.5. Background was subtracted using the median of the top and bottom of the band. STAT3 phosphorylation was calculated as phospho-STAT3-Y705 integrated intensity over total STAT3 integrated intensity and then set relative to a control as specified in the figure.

#### RNAi experiments

HeLa cells at 37,500 in 24-well TC-treated plates were treated for 24 h with 5picomoles/well total siRNA from either non-targeting siGENOME (Dharmacon) siRNA #5 (NT5; #D-001210-05) or SMARTpool directed against human *SOCS3* (#M-004299-02-0005) and human *PTPN11* (#M-003947-01-0005) using Lipofectamine RNAiMAX (ThermoFisher, 13778075) Infections were then conducted 48 h after initial addition of siRNA as described above. Knockdown was confirmed in each experiment by qPCR following the same ΔΔCT method used for measuring expression of *TNIP3* and *SBNO2*.

#### Thermal shift assay

5 μM STAT3^136−705^ (purified as described in Gibbs et al., 2020^6^) was incubated with 25μM phosphopeptides (Genscript, Piscataway, New Jersey) from the binding sites of gp130 (SGpYRHQVPSV), SarA (SGpYIAQYRHS), SarA^I168R^ (SGpYRAQYRHS) and gp130^R168I^ (SGpYIHQVPSV) at room temperature for 5 min in an assay buffer containing 10 mM Tris-HCl, pH 8.0; 50 mM NaCl; 10% glycerol; 5 mM β-mercaptoethanol. Water was used as the vehicle control. Subsequently, the STAT3 and peptide mixtures were added to 1X Glomelt (Biotium #33021-1, Fremont, California) a dye that binds hydrophobic residues and fluoresces upon protein denaturation due to an increase in heat. The combined assay volume per well was 20 μL. There were 3 replicates per peptide per plate. STAT3 thermal shift assays (TSA) were performed in a 96-well PCR plate (Hard-shell and thin wall PCR plates, Bio-Rad, Hercules, California). Temperature gradient from 25°C to 95°C (increasing ramp rate of 0.05°C/s) was completed in a Bio-Rad CFX Connect Real-Time System, following the general guidelines in the Glomelt kit protocol. The slope of the first derivative of the fluorescence vs. temperature curve was used to calculate the melting temperature (Tm).

#### Mass spectrometry

Immunoprecipitates were adjusted to 5% (w/v) SDS and 50 mM triethylammonium bicarbonate (TEAB), pH 8.5, reduced with 10 mM dithiothreitol at 80°C for 10 min and alkylated with 25 mM iodoacetamide for 30 min and room temp followed by acidification with 1.2% (v/v) phosphosphoric acid and addition of 6 volumes of 90:10 MeOH:50 mM TEAB (binding/wash buffer). Solutions were loaded onto an S-Trap micro column (Protifi) at *4000* xg for 30 s followed by washing with 4 x 150 μL of wash binding buffer. Digestions were performed with 1 μg of wild type alpha lytic protease (Cell Signaling Technology #33036) in 25 μL TEAB for 1.5 h at 47°C followed by elution with 40 μL of TEAB, 40 μL of 99.8/0.2 (v/v) H_2_O:formic acid and 35 μL of 50/49.8/0.2 (v/v/v) MeCN: H_2_O:formic acid. Eluents were lyophilized and reconstituted in 20 μL of 0.1% formic acid, and 7.5 μL of each sample was loaded onto Evotip Pure disposable C18 trap columns and analyzed using an Evosep One LC interfaced to an Thermofisher Orbitrap Astral MS. Each sample was analyzed twice using a 30 sample-per-day method and MS/MS data-dependent (DDA) and data-independent acquisition (DIA) as shown in Table S6. Database searching using a Uniprot human database (downloaded on 08/17/24) appended with transgenes and common contaminants (20,472 total entries) was performed with Spectronaut 19.2 using the Pulsar search engine with semispecific WALP cleavage specificity (cleavage C-terminal to T,A,S or V with non-specific cleavage on either C- or N terminus) and variable phosphorylation on S/T/Y. The DIA acquisition was used for identification and quantification of peptide precursors and protein groups at a 1% false discovery rate in Spectronaut. Local normalization was applied,^47^ and protein group quantities were determined using the MaxLFQ algorithm.^48^ Phosphorylation site localization used a probability cut-off of 0.75.^49^

#### Kinase assay

GSK-3α/β−/− 293ET cells (Panagi et al., 2020) seeded in a 6-well format were transiently transfected with ptCMV.GFP, ptCMV.GFP-SarAΔΝ44 or ptCMV.GFP-SarAΔΝ44^I168R^ for 48 h. The sequence encoding the N-terminal 44 amino acid residues of SarA were deleted, as they do not appear to affect activity, and this removes an additional tyrosine phosphorylation site to simplify interpretation. 800 ng of plasmid was transfected in each well of cells and four wells were used per condition. Post-transfection, cells from the same transfection condition were pooled together and lysed in 1.4 mL Lysis Buffer (150 mM NaCl, 0.3% Triton X-100, 20 mM Tris-Cl pH 7.4, 5% Glycerol, 5 mM EDTA), supplemented with a Protease Inhibitor Cocktail (cOmplete, Roche), and clarified by centrifugation at 17,000 x g for 10 min at 4°C. Thereafter, GFP-tagged proteins were enriched on beads by GFP-immunoprecipitation. Briefly, GFP-Trap agarose beads (ChromoTek) equilibrated in cold lysis buffer were incubated with the post-nuclear supernatant fraction for 3–5 h at 4°C with rotation. Post-incubation, beads were washed three times in 1 mL Lysis Buffer and twice in 1 mL 1x Kinase Buffer (Cell Signaling, 9802) supplemented with Phosphatase Inhibitors (PhosSTOP, Roche). Beads containing GFP-tagged targets were distributed evenly in kinase reaction tubes and resuspended in 50 μL 1x Kinase Buffer containing 1 mM ATP (ThermoFisher) with or without 0.2 μM of non-phosphorylated Avi-GSK-3βS9A and 0.2 μM His6-STAT3127-715 (Panagi et al., 2020). Kinase reactions were carried out at 30°C with agitation at 600 RPM in a PCMT Grant-bio Thermo-Shaker and stopped at the indicated time point by adding 5x SDS loading buffer (312.5 mM Tris-Cl pH 6.8, 10% SDS, 25% Glycerol, Bromophenol Blue) containing 20% β-Mercaptoethanol. The samples were then boiled at 95°C for 7 min, centrifuged at 1500 x g for 1 min and eluted proteins were subjected to immunoblot analysis.

#### Multiple sequence comparison

*Salmonella* genomes deposited in the PubMLST database (*n* = 21,223) were tested for presence of sarA by conducting BLASTp pairwise alignment in PubMLST.^26^ Sequences from samples with a resulting sarA alignment length of >100 residues and a BLAST E-value <0.001 were aligned using the CLUSTAL W algorithm^50^ executed through the R package *msa* v.1.34.0.^51^ Mean BLASTp E-values from serovars containing samples passing the above criteria were merged with an existing *Salmonella* maximum-likelihood phylogenetic tree described in^27^ using the R packages *treeio* v.1.26.0^52^ and *ggtree* v.3.10.0.^53^ Protein sequences of IL6ST and GSK-3β homologs from Ensembl^54^ were aligned using the CLUSTAL W algorithm as described above. Sequence logos were generated using the R package *ggseqlogo* v.0.1.^45^

### Quantification and statistical analysis

Descriptive statistics were performed with GraphPad Prism v10 (GraphPad Software, US) or R v4.3.1 (R Core Team) using tidyverse,^55^ magrittr,^56^ ggpubr,^57^ and venn^58^ packages. The size of each study or number of replicates, along with the statistical tests performed can be found in figure legends. *In vitro* inter-experimental variability was removed prior to data visualization or statistical analysis by making each experimental mean equal to the grand mean of all experiments combined. This is done by multiplying all values within each experiment by a normalization constant. The normalization constant is calculated by dividing the grand mean of all combined experiments by the mean of each specific experiment. Bar graphs represent the mean ± SEM (standard error of mean), unless otherwise noted. The variability of the data are represented by plotting the individual sample points on each of our graphs.

## Supplemental information

Document S1. Figures S1–S7 and Tables S3–S6

Table S1. Excel file containing results from SarA IP/MS, related to Figure 4 (A) Tab in excel file containing protein abundances. (B) Tab in excel file containing peptide abundances

Table S2. Excel file containing multiple sequence alignment related to Figure 6 (A) Tab in excel file containing MSA of SarA homologs. (B) Tab in excel file containing MSA of GSK3β homologs. (C) Tab in excel file containing MSA of IL6ST homologs

Document S2. Article plus supplemental information
