# Supplementary material for: A single amino acid in the Salmonella effector SarA/SteE triggers supraphysiological activation of STAT3 for anti-inflammatory gene expression
Source: Cell Rep. 2025 Apr 5;44(4):115530. doi: 10.1016/j.celrep.2025.115530 (PMC12014907; doi:10.1016/j.celrep.2025.115530)
Supplement: Document S1. Figures S1–S7 and Tables S3–S6 [file mmc1.pdf]

Cell Reports, Volume *44*

Supplemental information

A single amino acid in the *Salmonella* effector SarA/SteE triggers supraphysiological activation of STAT3 for anti-inflammatory gene expression

Margaret R. Gaggioli, Angela G. Jones, Ioanna Panagi, Erica J. Washington, Rachel E. Loney, Janina H. Muench, Matthew W. Foster, Richard G. Brennan, Teresa L.M. Thurston, and Dennis C. Ko

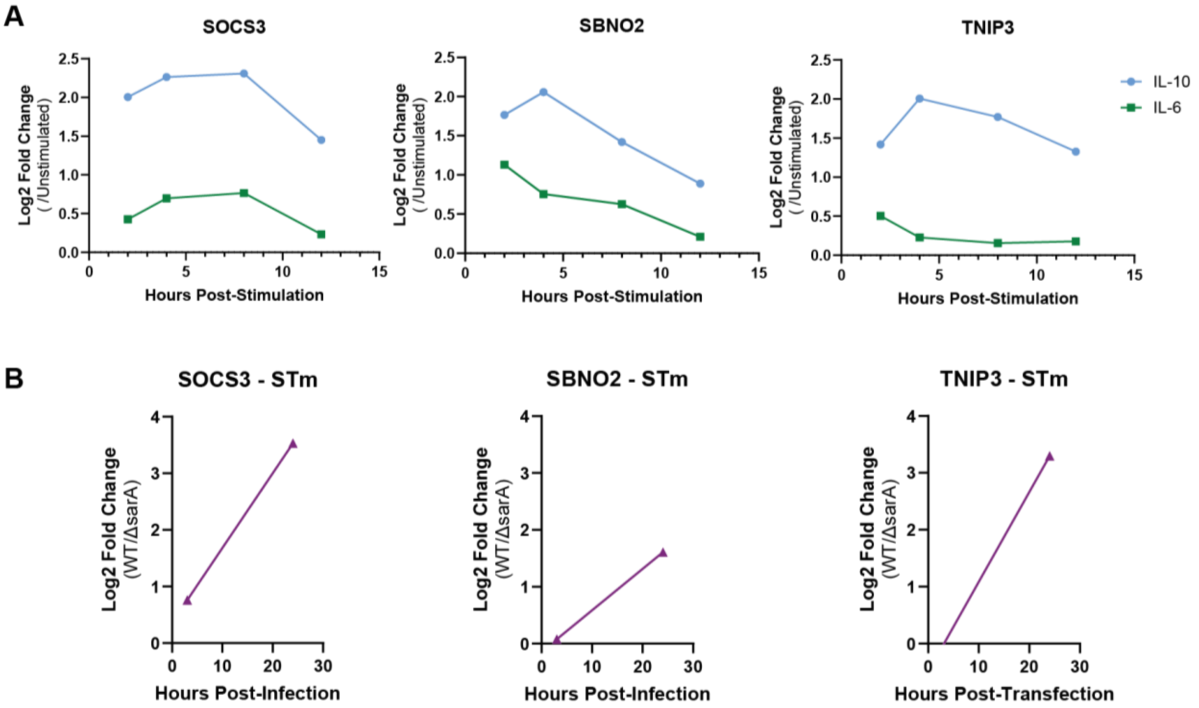

Figure S1: Expression of anti-inflammatory genes after IL10 stimulation, IL-6 stimulation, or *S.* Typhimurium infection. Related to Fig. 1B. Log2-fold changes of *SOCS3*, *SBNO2*, and *TNIP3* are from previously published transcriptomic datasets using (A) IL-6 or IL-10 stimulation (Braun et al., 2013) and (B) infection with wild-type or Δ*sarA S.* Typhimurium (Jaslow et al., 2018).

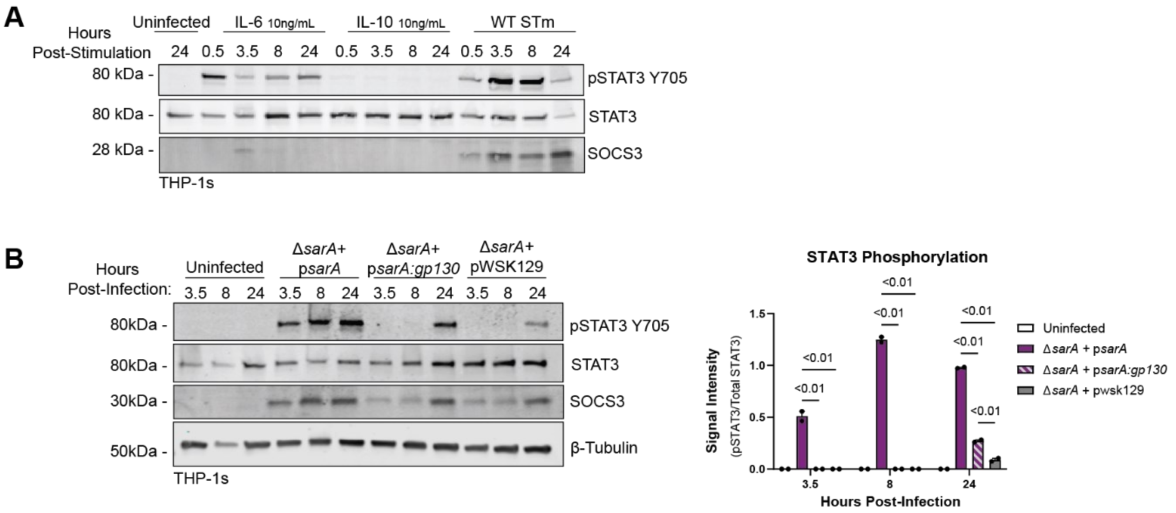

Figure S2: THP-1 cells have minimal STAT3 phosphorylation in response to IL-10 stimulation and Δ*sarA* infection. Related to Fig. 1. (A) Western blot from THP-1 monocytes that were stimulated for the time indicated with IL-6, IL-10, or infected with wild-type *S.* Typhimurium at MOI10. (B) Western blot of THP-1 monocytes that were infected with either Δ*sarA* + pWSK129, Δ*sarA* + p*sarA,* or Δ*sarA* + p*sarA:gp130* at MOI10 for indicated timepoints. Graph shows STAT3 phosphorylation quantified as the signal intensity of pSTAT3 bands over the signal intensity of the total STAT3 bands from two experiments. Data represented as mean +/- SEM. P-values obtained from two-way ANOVA followed by Tukey's multiple comparison's test.

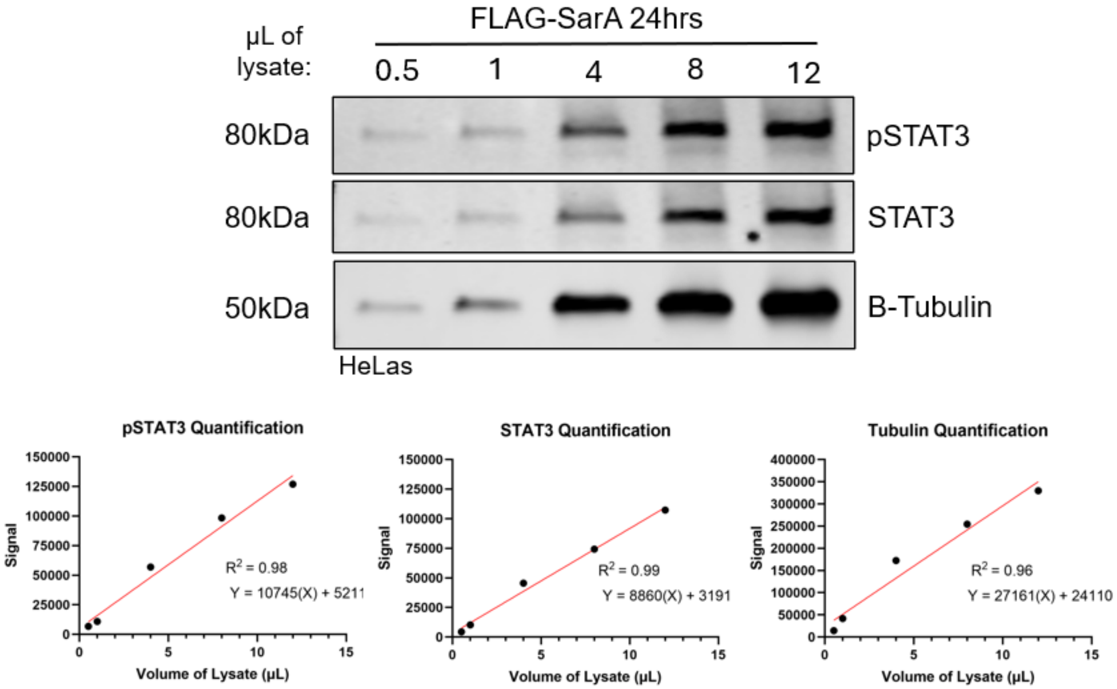

Figure S3: Detected phospho-STAT3, STAT3, and β-tubulin were all in the linear range of the assay. Related to Fig. 1. Cell lysates from HeLa cells overexpressing FLAG-SarA were loaded on a protein gel in various dilutions. The designated μL of sample lysates were all brought up to a volume of 12μL with 1x SDS-PAGE loading buffer and then loaded into a protein gel. Quantification of bands in western blots were conducted using near-infrared secondary antibodies and quantification using a LiCOR Odyssey.

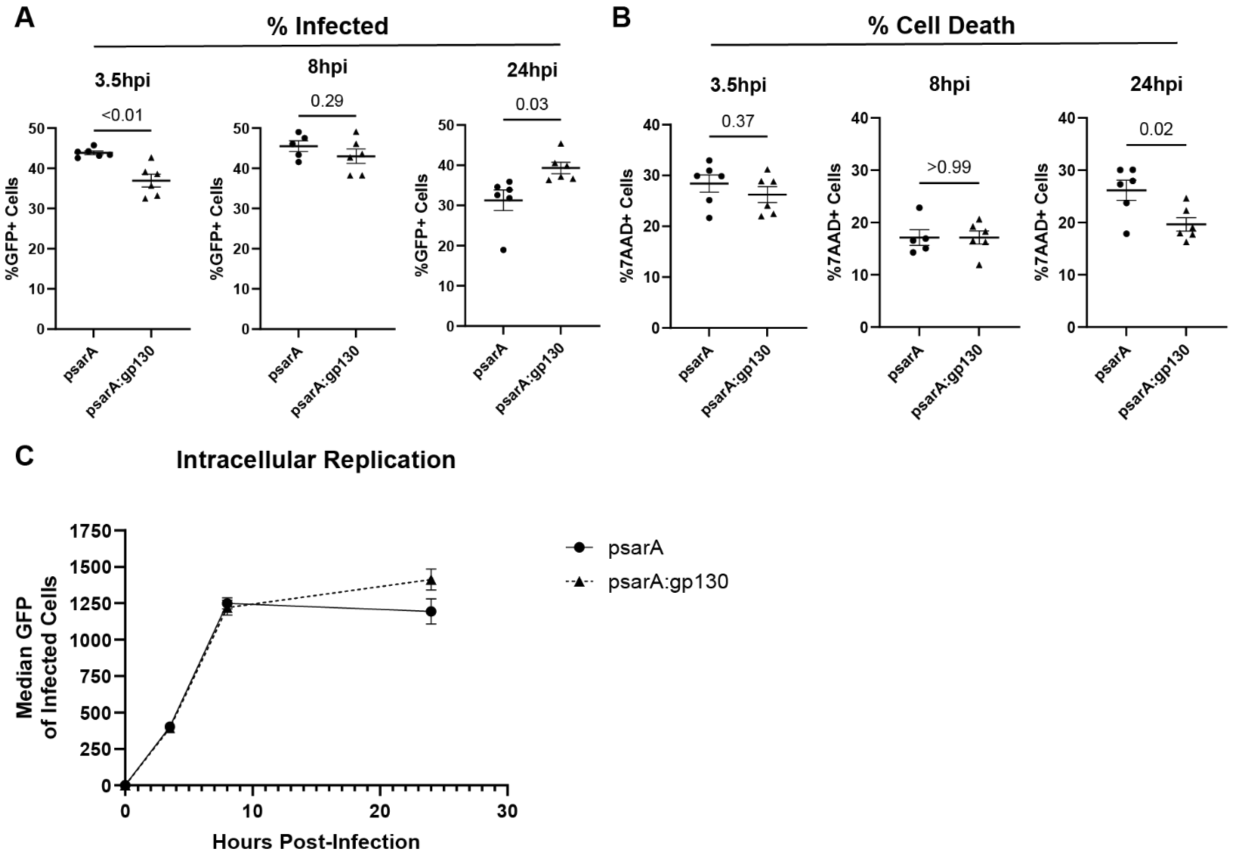

Figure S4: *sarA* and *sarA:gp130* complemented strains of *S.* Typhimurium have comparable infection and intracellular growth in THP-1 monocytes. Related to Fig. 1H-J. THP-1 monocytes were infected with inducible GFP expressing *S.* Typhimurium strains. Flow cytometry was used to measure the (A) percentage of GFP+ cells at three different timepoints as a readout of % infection. (B) Before each timepoint, cells were stained with 7AAD and the percentage of 7AAD+ cells was used as a readout of % cell death. For A and B, points represent six biological replicates across three independent experiments. Data represented as mean +/- SEM. P-values obtained from Welch's t-tests. (C) Intracellular bacterial replication as measured by median GFP value of GFP+ cells was measured across three timepoints.

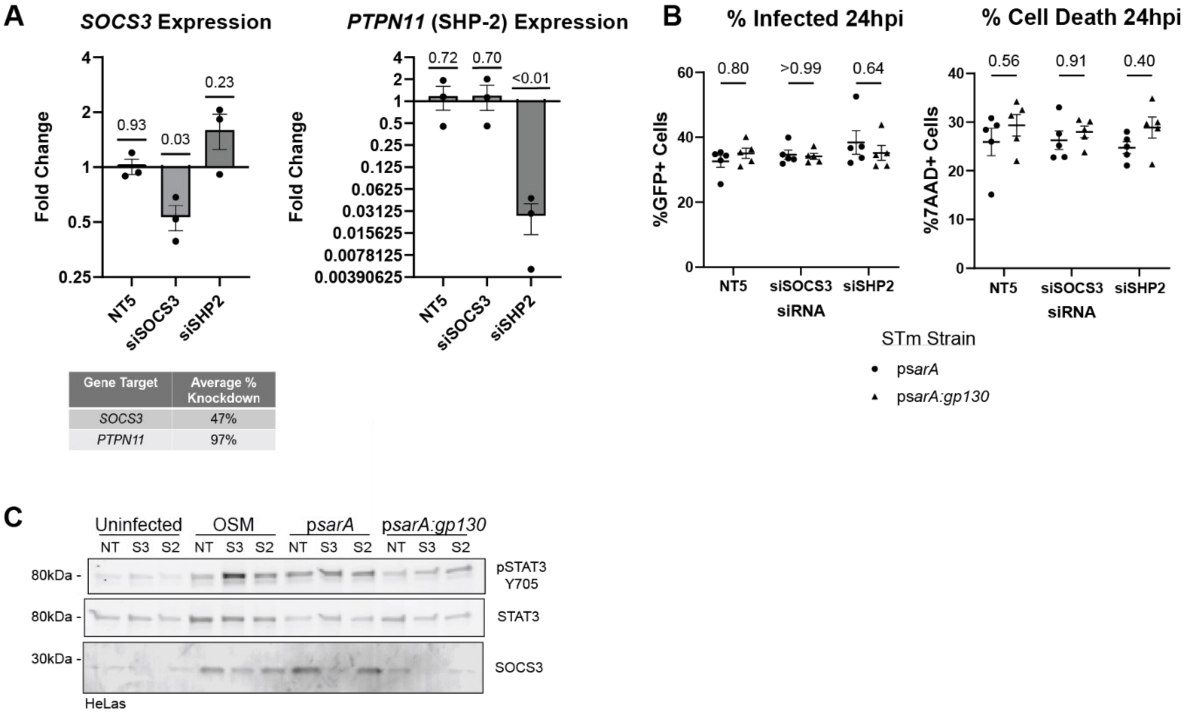

Figure S5: Knockdown of *SOCS3* and *PTPN11* (SHP-2) have no significant effect on SarA directed STAT3 phosphorylation. Related to Fig. 2. (A) qPCR data confirming transcriptional knockdown of *SOCS3* and *PTPN11* in uninfected samples from three experiments. Data represented as mean +/- SEM. P-values obtained from one sample t-tests. (B) HeLa cells were infected with inducible GFP expressing *S.* Typhimurium strains. Flow cytometry was used to measure the percentage of GFP+ cells at three different timepoints as a readout of % infection. Before each timepoint, cells were stained with 7AAD and the percentage of 7AAD+ cells was used as a readout of % cell death. Points represent five biological replicates across five independent experiments. Data represented as mean +/- SEM. P-values obtained from 2-way ANOVA with Sidak's multiple comparison test. (C) Western blot of cell lysates showing that infection with *sarA:gp130* complemented strain of *S.* Typhimurium leads to greater pSTAT3 levels than in uninfected cells.

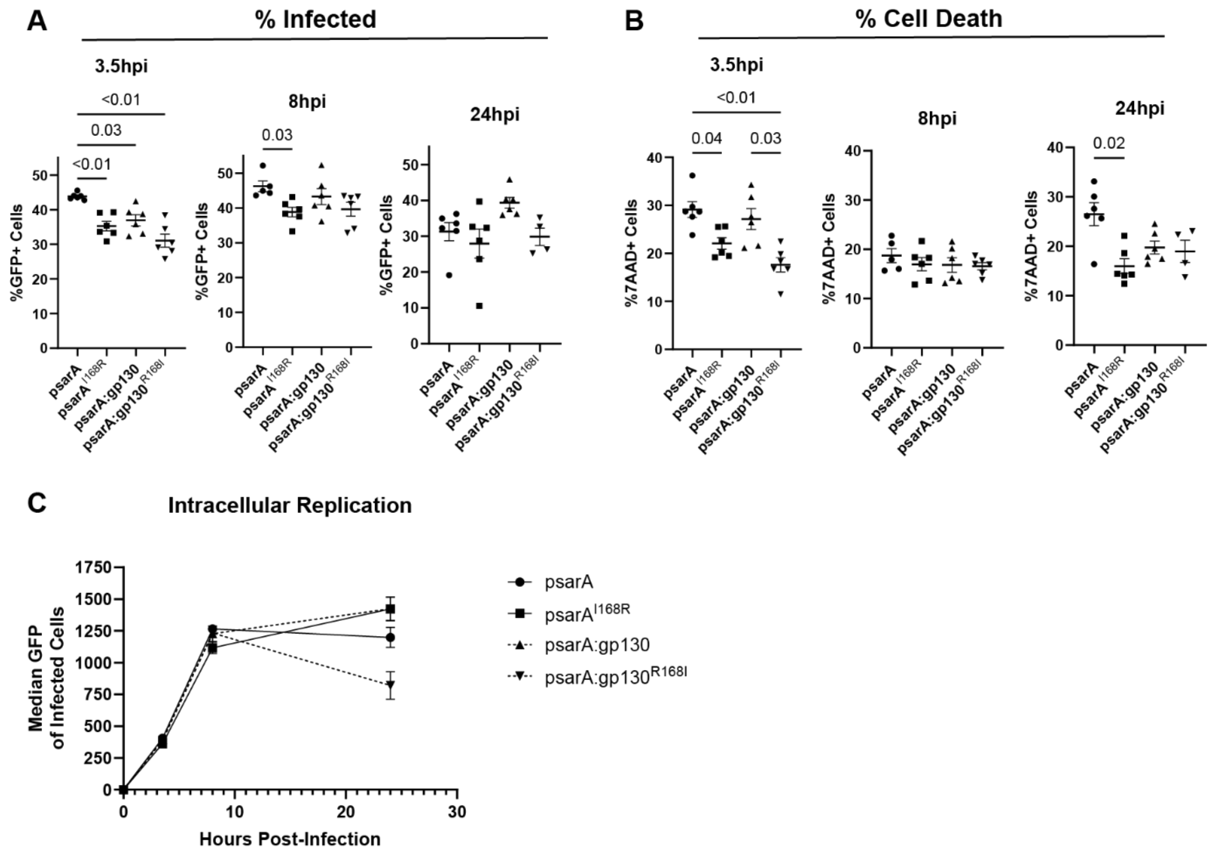

Figure S6: *sarA sarA*^{I168R}, *sarA:gp130*, and *sarA:gp130*^{R168I} complemented strains of *S.* Typhimurium have comparable infection and intracellular growth in THP-1 monocytes. Related to Fig. 3C. THP-1 monocytes were infected with inducible GFP expressing *S.* Typhimurium strains. Flow cytometry was used to measure the (A) percentage of GFP+ cells at three different timepoints as a readout of % infection. (B) Before each timepoint, cells were stained with 7AAD and the percentage of 7AAD+ cells was used as a readout of % cell death. For A and B, points represent six biological replicates across three independent experiments. Data represented as mean +/- SEM. P-values obtained from Brown-Forsythe and Welch ANOVA followed by Dunnett's T3 multiple comparisons test. (C) Intracellular bacterial replication as measured by median GFP value of GFP+ cells was measured across three timepoints.

```
FLAG-SarA                 150 ISSNDECKLSGSTSCTTPASHNPPSGYIAQYRHSAEVFPDE 190
                                                 SHNPPSGYIA (pTyr)
                                                 SHNPPSGYIA

FLAG-SarA_I168R           150 ISSNDECKLSGSTSCTTPASHNPPSGYRAQYRHSAEVFPDE 190
                                                 SHNPPSGYRA
                                                  HNPPSGYRA
                                                  HNPPSGYRAQYRHS
                                                   NPPSGYRAQYRHS
                                                    PPSGYRAQYRHS
                                                      SGYRAQYRHS

FLAG-SarA:gp130           150 ISSSDENESSQNTSSTVQYSTVVHSGYRHQVPSVQVFPDE 189
                                                     HSGYRHQVPS
                                                      SGYRHQVPS
                                                       GYRHQVPS
                                                       GYRHQVP

FLAG-SarA:gp130_R168I     150 ISSSDENESSQNTSSTVQYSTVVHSGYIHQVPSVQVFPDE 189
                                                    VHSGYIHQV
                                                     HSGYIHQVPS (pTyr)
                                                     HSGYIHQVPS
                                                       GYIHQVPSV
                                                       GYIHQVPS
                                                        YIHQVPSV
                                                        YIHQVPS
```

Figure S7: Mass spectrometry analysis of SarA and SarA:gp130 chimeras. Related to Fig. 4B. Flag immunoprecipitates were digested with wild-type alpha lytic protease followed by LC-MS/MS. Variable sequence domains (amino acids 150-189/190) are shown for each construct, with peptides identified by LC-MS/MS shown for each construct. Identified sites of phosphorylation are shown in red.

Plasmid	Resistance	In E. coli Strain	Notes
pEGFP-C1	Kan	DCK53	
pcDNA3	Amp	DCK77	
pcDNA3-FLAG-*sarA*	Amp	DCK796	Gibbs et al.,2020
pcDNA3-FLAG-*sarA*^{YSTV}	Amp	DCK1150	
pcDNA3-FLAG-*sarA*^{I168R}	Amp	DCK1201	
pcDNA3-FLAG-*sarA*^{A169H}	Amp	DCK1202	
pcDNA3-FLAG-*sarA*$^{IA\text{->}RH}$	Amp	DCK1203	
pcDNA3-FLAG-*sarA*^{I169L}	Amp	DCK1204	
pcDNA3-FLAG-*sarA:gp130*	Amp	DCK801	Gibbs et al.,2020
pcDNA3-FLAG-*sarA:gp130*^{Y159F}	Amp	DCK1151	
pcDNA3-FLAG-*sarA:gp130*^{R168I}	Amp	DCK1205	
pcDNA3-FLAG-*sarA:gp130*$^{RH\text{->}IA}$	Amp	DCK1206	
pcDNA3-FLAG-*gp130dimer*	Amp	DCK824	Gibbs et al.,2020
pcDNA3-FLAG-*gp130dimer*$^{R\text{->}I}$	Amp	DCK1254	
pcDNA3-FLAG-*gp130dimer:sarA*	Amp	DCK825	Gibbs et al.,2020
p67GFP3.1	Amp		Pujol & Bliska, 2003
pWSK129	Kan	DCK827	Wang & Kushner, 1991
pWSK129-*sarA*	Kan	DCK809	
pWSK129-*sarA*^{I168R}	Kan	DCK1221	
pWSK129-*sarA:gp130*	Kan	DCK852	
pWSK129-*sarA:gp130*^{R168I}	Kan	DCK1220	
ptCMV.GFP			From Thurston Lab, Panagi et al., 2020
ptCMV.GFP-*SarA∆20*			From Thurston Lab, Panagi et al., 2020
ptCMV.GFP-*SarA∆20*^{I168R}			From Thurston Lab

Table S3: Plasmids. Related to STAR★METHODS.

Designation	Name	Purpose	Sequence
DK710	sarA-gp130_Y759F_Fwd	Y759F in codon-opt *sarA:gp130*	5'-gcaccacggtggagaactgcactgtgcta-3'
DK711	sarA-gp130_Y759F_Rvr	Y759F in codon-opt *sarA:gp130*	5'-tagcacagtgcagttctccaccgtggtgc-3'
DK793	sarA_A159Y_H161T_N162V_Fwd	ASHN → YSTV in codon-opt *sarA*	ctgagcaatgtagccgcttgggggga**AC**a**GT**gct**ATA**gggtgtggtacaactagtagagcc
DK794	sarA_A159Y_H161T_N162V_Rvr	ASHN → YSTV in codon-opt *sarA*	ggctctactagttgtaccacaccc**TAT**agc**AC**t**GT**tcccccaagcggctacattgctcag
DK1127	FLAG_SarA_pY-1_R_fwd	I168R in codon-opt *sarA*	gccgatactgagccctgtagccgcttggggga
DK1128	FLAG_SarA_pY-1_R_rev	I168R in codon-opt *sarA*	tcccccaagcggctacagggctcagtatcggc
DK1129	FLAG_SarA_pY-2_H_fwd	A169H in codon-opt *sarA*	gtgccgatactgatgaatgtagccgcttgggggga
DK1130	FLAG_SarA_pY-2_H_rev	A169H in codon-opt *sarA*	tcccccaagcggctacattcatcagtatcggcac
DK1131	FLAG_SarA_pY-1-2_RH_fwd	Y**IA**Q → Y**RH**Q in codon-opt *sarA*	gcgctgtgccgatactgatgcctgtagccgcttgggggattatg
DK1132	FLAG_SarA_pY-1-2_RH_rev	Y**IA**Q → Y**RH**Q in codon-opt *sarA*	cataatcccccaagcggctacaggcatcagtatcggcacagcgc
DK1133	FLAG_SarA_pY-1_L_fwd	I168L in codon-opt *sarA*	ccgatactgagcaaggtagccgcttgggg
DK1134	FLAG-SarA_pY-1_L_rev	I168L in codon-opt *sarA*	ccccaagcggctaccttgctcagtatcgg
DK1135	FLAG_SarA:gp130_pY-1_I_fwd	R168I in codon-opt *sarA:gp130*	cggagggcacctggtgtatatagccagagtgcaccac
DK1136	FLAG_SarA:gp130_pY-1_I_rev	R168I in codon-opt *sarA:gp130*	gtggtgcactctggctatatacaccaggtgccctccg
DK1020	sarA-gp130_R168I_H169A_F	Y**RH**Q → Y**IA**Q in codon-opt *sarA :gp130*	ggagggcacctgggctatatagccagagtgcaccacggtggag
DK1021	sarA-gp130_R168I_H169A_R	Y**RH**Q → Y**IA**Q in codon-opt *sarA :gp130*	ctccaccgtggtgcactctggctatatagcccaggtgccctcc
DK1141	psk129_sarA_t503g_c504g_fwd	I168R in native *sarA*	atgcctgtattgagccctataaccggacggtgggttatg
DK1142	psk129_sarA_t503g_c504g_rev	I168R in native *sarA*	cataacccaccgtccggttatagggctcaatacaggcat
DK1143	pwsk129_sarA_g505c_c506a_fwd	A169H in native *sarA*	gcagaatgcctgtattgatggatataaccggacggtgg
DK1144	pwsk129_sarA_g505c_c506a_rev	A169H in native *sarA*	ccaccgtccggttatatccatcaatacaggcattctgc
DK1145	pwsk129_sarA:gp130_c502a_g503t_fwd	Y**RH**Q → Y**IA**Q in *sarA :gp130*	ggaacctggtgaatataaccgctgtgaaccacggtgc
DK1146	pwsk129_sarA:gp130_c502a_g503t_rev	Y**RH**Q → Y**IA**Q in *sarA :gp130*	gcaccgtggttcacagcggttatattcaccaggttcc

DK1170	FLAG-gp130_R238I_fwd	R238I in codon-opt *gp130dimer*	5'-ggcacctggtgtatatagccggagtgcaccacg-3'
DK1171	FLAG-gp130_R238I_rev	R238I in codon-opt *gp130dimer*	5'-cgtggtgcactccggctatatacaccaggtgcc-3'
Trx651	SarAΔN20_fwd	Clamp, PciI, SarAΔN20	cgcgggacatgtca GATGTTAATTTAGAGGAC
IOP197	SarAΔN20_I168R_rev	Clamp, NotI, stop codon, SarA with I68R mutation	cgcgggGCGGCCGCTTATTCATCCGGGAAAACCTCTGCAGAATGCCTGTATTGAGCGCGATAACCGGACGGTGG

Table S4: Primers. Related to STAR★METHODS.

Strain	Genotype	Plasmid	Resistance	Derived From	Notes
DCK22	14028s	p67GFP	Amp	CS093	
DCK444	14028s Δ*sarA*	P67GFP	Amp	DCK440 (14028s Δ*sarA)*	Jaslow et al., 2018
DCK486	14028s Δ*sarA*	pWSK129	Kan	DCK444	Jaslow et al., 2018
DCK487b	14028s Δ*sarA*	pWSK129-*sarA*, p67GFP	Amp, Kan	DCK444	Jaslow et al., 2018
DCK1156	14028s Δ*sarA*	pWSK129-*sarA:gp130*, p67GFP	Amp, Kan	DCK869 (14028s Δ*sarA*+ pWSK129-*sarA:gp130*)	
DCK1222	14028s Δ*sarA*	pWSK129-*sarA*^{I168R}, p67GFP	Amp, Kan	DCK1224 (14028s Δ*sarA*+ pWSK129-*sarA*^{I168R})	
DCK1223	14028s Δ*sarA*	pWSK129-*sarA:gp130*^{R168I}, p67GFP	Amp, Kan	DCK1225 (14028s Δ*sarA*+ pWSK129-*sarA:gp130*^{R168I})	

Table S5: Bacterial Strains. Related to STAR★METHODS.

**Method**	**Data-dependent acquisition (DDA)**	**Data-independent acquisition (DIA)**
MS1 resolution	120,000 (Orbitrap)	240,000 (Orbitrap)
MS1 range (m/z)	375-1500	380-980
MS1 automatic gain control	300%	500%
MS1 max injection time	50 ms	50 ms
Cycle time	1.5 s	0.6 s
MS2 precursor mass range (m/z)	375-1500	380-980
MS2 scan range (m/z)	100-1200 (Astral)	150-2000 (Astral)
MS2 window width	1.2 m/z	4 m/z
MS2 automatic gain control	standard	500%
MS2 max injection time	10 ms	6 ms
MS2 normalized collision energy	28%	28%

Table S6: Mass Spectrometry Method Settings. Related to STAR★METHODS.

Braun, D.A., Fribourg, M., and Sealfon, S.C. (2013). Cytokine response is determined by duration of receptor and signal transducers and activators of transcription 3 (STAT3) activation. J Biol Chem *288*, 2986-2993.

Jaslow, S.L., Gibbs, K.D., Fricke, W.F., Wang, L., Pittman, K.J., Mammel, M.K., Thaden, J.T., Fowler, V.G., Jr., Hammer, G.E., Elfenbein, J.R.*, et al.* (2018). Salmonella Activation of STAT3 Signaling by SarA Effector Promotes Intracellular Replication and Production of IL-10. Cell Rep *23*, 3525-3536.

Gibbs, K.D., Washington, E.J., Jaslow, S.L., Bourgeois, J.S., Foster, M.W., Guo, R., Brennan, R.G., and Ko, D.C. (2020). The Salmonella Secreted Effector SarA/SteE Mimics Cytokine Receptor Signaling to Activate STAT3. Cell Host Microbe *27*, 129-139 e124. 10.1016/j.chom.2019.11.012.
